# Evaluating the Compatibility of New Recombinant Protein Antigens (Trivalent NRRV) with a Mock Pentavalent Combination Vaccine Containing Whole-Cell Pertussis: Analytical and Formulation Challenges

**DOI:** 10.3390/vaccines12060609

**Published:** 2024-06-03

**Authors:** Prashant Kumar, David A. Holland, Kathryn Secrist, Poorva Taskar, Brandy Dotson, Soraia Saleh-Birdjandi, Yetunde Adewunmi, Jennifer Doering, Nicholas J. Mantis, David B. Volkin, Sangeeta B. Joshi

**Affiliations:** 1 Department of Pharmaceutical Chemistry, Vaccine Analytics and Formulation Center, University of Kansas, Lawrence, KS 66047, USA; 2 New York State Department of Health, Division of Infectious Diseases, Wadsworth Center, Albany, NY 12208, USA

**Keywords:** pediatric combination vaccine, aluminum-salt adjuvant, non-replicating rotavirus vaccine, diphtheria, tetanus, whole-cell pertussis, hepatitis B, *Haemophilus influenzae*, formulation, compatibility, stability, preservatives

## Abstract

Introducing new recombinant protein antigens to existing pediatric combination vaccines is important in improving coverage and affordability, especially in low- and middle-income countries (LMICs). This case-study highlights the analytical and formulation challenges encountered with three recombinant non-replicating rotavirus vaccine (NRRV) antigens (t-NRRV formulated with Alhydrogel^®^ adjuvant, AH) combined with a mock multidose formulation of a pediatric pentavalent vaccine used in LMICs. This complex formulation contained (1) vaccine antigens (i.e., whole-cell pertussis (wP), diphtheria (D), tetanus (T), *Haemophilus influenza* (Hib), and hepatitis B (HepB), (2) a mixture of aluminum-salt adjuvants (AH and Adju-Phos^®^, AP), and (3) a preservative (thimerosal, TH). Selective, stability-indicating competitive immunoassays were developed to monitor binding of specific mAbs to each antigen, except wP which required the setup of a mouse immunogenicity assay. Simple mixing led to the desorption of t-NRRV antigens from AH and increased degradation during storage. These deleterious effects were caused by specific antigens, AP, and TH. An AH-only pentavalent formulation mitigated t-NRRV antigen desorption; however, the Hib antigen displayed previously reported AH-induced instability. The same rank-ordering of t-NRRV antigen stability (P[8] > P[4] > P[6]) was observed in mock pentavalent formulations and with various preservatives. The lessons learned are discussed to enable future multidose, combination vaccine formulation development with new vaccine candidates.

## 1. Introduction

By introducing combination vaccines into routine pediatric immunization schedules, numerous public health and economic benefits have been achieved including enhanced disease protection and reduced costs [1,2]. Combination vaccines provide additional societal benefits for families and healthcare providers including improved compliance (i.e., completion of the entire vaccination series) and higher productivity (i.e., fewer visits save time and money) [2]. Overall, worldwide implementation of combination vaccines has enhanced global vaccine access and immunization coverage, especially in low- and middle- income countries (LMICs) [3,4].

The pediatric combination vaccine comprising diphtheria and tetanus toxoids and inactivated whole-cell pertussis bacteria (DTwP) has played an important role in protecting the global pediatric population for over half a century [1]. Although the reactogenicity profile of this vaccine has been improved by replacing wP with aP (acellular pertussis) antigens, the latter vaccine has primarily been used in high-income countries due to (1) the high cost of manufacturing five different aP antigens [1] and (2) the observation that vaccines containing aP antigens display more rapid waning of immunity against pertussis infection compared to vaccines containing the wP antigen [4,5]. Based on these considerations, pediatric combination vaccines containing wP are primarily used in LMICs and are formulated as multidose presentations to further lower costs [5]. Typically, thimerosal (TH) is used as a preservative in the final drug product since TH is also part of the wP inactivation process used during commercial bulk manufacturing [6].

The addition of the *Haemophilus influenzae* (Hib) antigen (comprising polyribosyl ribitol phosphate (PRP) chemically conjugated to tetanus toxoids) and the recombinant hepatitis B (Hep B) surface antigen (self-assembled into a virus-like particle) to the DTwP has resulted in quadrivalent (i.e., DTwP-Hib and DTwP-Hep B) and pentavalent (i.e., DTwP-Hib-HepB) formulations of pediatric combination vaccines, and their introduction has helped to improve immunization compliance in pediatric and adult populations worldwide [1,7,8,9]. The successful introduction of additional new vaccine antigens into these quadrivalent and/or pentavalent combination vaccines, however, has historically been a long and time-consuming process taking decades of development work to implement. Its challenges include formulation complexity leading to compatibility and stability issues, manufacturing complexity leading to the need for strict and expensive quality control monitoring, and clinical complexities caused by immunological interferences leading to different antigen doses and injection schedules [2].

Despite such technical hurdles, public health and societal benefits for LMICs provide strong incentives to add new antigens into the pediatric pentavalent combination vaccine (i.e., DTwP-Hib-HepB). For example, essentially every child by 5 years of age experiences a rotavirus (RV) infection which can cause severe gastroenteritis and diarrhea with potential life-threatening effects without timely medical care [10]. RV infection led to ~128,000 deaths in 2016 worldwide [10], despite the availability of RV vaccines for children (i.e., two FDA-approved vaccines, four WHO prequalified vaccines, and some additional locally approved RV vaccines), all consisting of live, attenuated RVs delivered orally [11,12]. The vaccine efficacy of live RV vaccines varies considerably, however, between LMICs (~40–60%) and high-income countries (~80–90%) [13,14,15,16]. Moreover, the overall success of a RV vaccine also relies on its global coverage, which is currently only about ~30% for live RV vaccines due to manufacturing and cost limitations. In summary, since current 1st generation oral, live RV vaccines have lower efficacy in LMICs and are expensive to produce with limited manufacturing capacity, the introduction of new injectable recombinant protein subunit RV vaccine antigens (combined with the currently used pediatric combination vaccine) offers the potential of greatly improved vaccine coverage irrespective of the socio-economic background of a child [17,18,19].

An example of a recombinant protein subunit RV vaccine candidate is the non-replicating rotavirus vaccine (NRRV) which contains a trivalent mixture of recombinant truncated VP8* fusion protein antigens [11]. The trivalent NRRV (t-NRRV) vaccine candidate consists of three NRRV antigens produced recombinantly in *E. coli* as fusion proteins and are named as P2-VP8-P[4], P2-VP8-P[6], and P2-VP8-P[8] where P2 refers to the tetanus toxoid epitope and VP8-P[x] represents the ΔVP8* protein derived from human RV strain DS-1 (G2P[4]), 1076 (G2P[6]), or Wa (G1P[8]) [20,21]. The t-NRRV antigens are formulated with an aluminum-salt adjuvant for parenteral administration. Although t-NRRV showed promising results in early clinical trials, the trial’s sponsor PATH recently announced disappointing results from a pivotal Phase 3 clinical trial in Africa [22]. Nonetheless, for this work, t-NRRV serves as a case-study of the analytical and formulation challenges encountered when attempting to expand the pediatric combination pentavalent (DTwP-Hib-Hep) vaccine currently used in LMICs to include new recombinant protein antigens.

The goal of this work was to evaluate the compatibility of aluminum-adjuvanted t-NRRV with a mock formulation of the pediatric pentavalent vaccine (DTwP-Hib-Hep). A companion paper will cover examining the possibility of adding t-NRRV to t-IPV vaccines for the preparation of a possible bivalent vaccine [23]. One major challenge for combination vaccine formulation studies is the development and implementation of analytical assays. To this end, we set up a series of competitive immunoassays for aluminum-salt-adjuvanted antigens (t-NRRV, D, T, Hib, and HepB) in terms of binding to antigen-specific mAbs. For wP, no such in vitro antibody binding assays are available, so we implemented a mouse immunogenicity assay (instead of the mouse challenge assay used as a quality control test, i.e., the Kendrick assay) [24]. After establishing the stability-indicating nature of these assays, formulation studies were performed, including compatibility and stability assessments after mixing the t-NRRV and pentavalent vaccine antigens together in terms of alum adsorption, antibody binding, and wP immunogenicity. In addition, preservatives are also required to develop multidose formulations of such combination vaccines to further ease cost [25]. To this end, we also examined the effects of eight different preservatives on t-NRRV stability and selected promising leads for future multidose formulation development work. Finally, the overall outcomes are discussed, and recommendations for future work are provided in the context of the analytical and formulation challenges encountered when adding new recombinant protein antigens to the current pentavalent (DTwP-Hib-Hep) pediatric vaccine to improve affordability and global coverage in LMICs.

## 2. Materials and Methods

### 2.1. Materials

The three NRRV antigens (P[4], P[6], and P[8]) were kindly provided by SK Biologics (Seongnam-si, Republic of Korea) and PATH (Seattle, WA, USA). Diphtheria toxoid (D), tetanus toxoid (T), *Haemophilus influenzae* type b (Hib), and thimerosal (TH)-inactivated whole-cell pertussis (wP) antigens were purchased from Bio Farma (Jakarta Selatan, Indonesia), and the hepatitis B (Hep B) antigen was kindly provided by Biological E Ltd. (Hyderabad, India). Capture antibodies used in the ELISAs were obtained or purchased from various suppliers as described in detail in the results section. Aluminum hydroxide (Alhydrogel^®^, AH) and aluminum phosphate (Adju-Phos^®^, AP) adjuvants were purchased from InvivoGen (San Diego, CA, USA). The *Bordetella pertussis* type strain ATCC 18323 (American Type Culture Collection, Manassas, VA, USA) was used for the wP ELISA. The following antigens were used for antigen-specific Luminex assays: Adenylate Cyclase Toxin [ACT] and Pertactin [PRN] were obtained from List Biological Laboratories (Campbell, CA, USA), while filamentous hemagglutinin [FHA] and pertussis toxin [PT] were obtained from Sigma-Aldrich (St. Louis, MO, USA). All other reagents and chemicals used were of analytical grade or higher and purchased from Sigma-Aldrich (USA). Preservatives, namely, thimerosal, 2-phenoxyethanol, benzyl alcohol, phenol, and m-cresol, were purchased from Sigma-Aldrich (St. Louis, MO, USA), while chlorobutanol, methyl paraben, and propyl paraben were obtained from Spectrum Chemicals (Gardena, CA, USA).

### 2.2. Competitive ELISA Development for t-NRRV and Four Antigens (D, T, Hib, and HepB) in the Mock Pentavalent Combination

For the three NRRV antigens, the competitive ELISA has been described in detail elsewhere [26,27]. For the D, T, Hep, and Hib antigens, the ELISAs were adapted from previous reports but modified for use with aluminum-salt-adjuvanted samples [28,29,30,31]. Briefly, for each ELISA, the AH-adsorbed antigen was blocked using a casein blocking buffer (Thermo Fisher Catalog #37532 (Waltham, MA, USA)), followed by serial dilutions and incubation with a fixed amount of antigen-specific capture antibody overnight. The supernatant containing unbound specific capture antibody (as indicated in Table 1) was transferred to antigen-coated 96-well plates, followed by determining the amount of the bound antibody using tetramethylbenzidine (TMB) and recording the absorbance at OD450 using a SpectraMax^®^ ID5 plate reader (Molecular Devices, San Jose, CA, USA). The data were analyzed using an inverse prediction method in Origin 2020 (OriginLab Corporation, Northampton, MA, USA). Each ELISA was used for measuring the total antigen (bound + unbound) in the whole drug product and percentage bound antigen by measuring the supernatant (unbound) and pellet (bound) fractions of the drug product after centrifugation. Selectivity and stability-indication of each ELISA was confirmed before performing the compatibility and stability studies. Selectivity was tested in the absence of any stress condition, by comparing a known concentration of each (1) alum-adjuvanted specific antigen (assay standard) with (2) alum-adjuvanted specific antigens in the presence of the non-specific antigens and (3) alum-adsorbed, non-specific antigens minus the specific antigen. The stability-indication of each ELISA was assessed by thermal stress exposure of alum-adjuvanted antigens to specific temperatures and times: the NRRV P[4], P[6], and P[8] antigens at 50 °C for 40 min; T antigen at 60 °C for 1 h; D and HepB antigens at 70 °C for 1 h; and Hib antigen at 80 °C for 1 h. The stress samples were compared to the control samples of the same formulation stored at 2–8 °C for the same time.

### 2.3. Mouse Immunogenicity Assay for wP

Mouse immunogenicity studies for measuring wP were conducted under strict compliance with the Wadsworth Center’s Institutional Animal Care and Use Committee (IACUC). Briefly, female BALB/c mice aged 6–8 weeks were obtained from Taconic Biosciences (Germantown, NY, USA) and housed under conventional, specific pathogen-free conditions. In separate experiments, mice (n = 8–10 per experimental group; n = 6 for control groups) were vaccinated on day 0 with mock wP-containing trivalent, pentavalent, and hexavalent formulations. Final volumes of 250 µL were injected via the intraperitoneal (I.P) route. Blood was collected from the mice via the submandibular vein on days 30 and 60. The resulting sera were analyzed using a combination of the wP ELISAs and multiplex immune assays, as described in detail below.

### 2.4. wP ELISA

Immulon 4HBX 96-well plates (Thermo Scientific, USA) were coated with 100 µL/well of the *B. pertussis* strain 18323 (ATCC code 9797) that was grown on Oxoid charcoal agar, resuspended in PBS, and adjusted to an OD_600_ of 0.1 (~1 × 10^8^ CFUs/mL). Plates were stored in a biosafety hood overnight to allow the liquid phase to evaporate. Once fully dry, plates were blocked for 2 h with a 2% goat serum (Gibco, Gaitherberg, MD, USA) in PBS with 0.05% Tween-20 (PBS-T) at room temperature. The serum samples were diluted in a block solution at 1:100 in a separate PVC plate. The dilutions were transferred to the wP-coated microtiter plates, and 3-fold serial dilutions were performed. The plates were incubated with the serum for 1 h at room temperature before being washed three times. Next, 100 µL/well of goat anti-mouse IgG-HRP (Southern Biotech, Birmingham, AL, USA) at 1:2000 was applied to all the plates for 30 min. The plates were washed again as described above, and 100 µL/well of TMB was added for 4.5 min. The reaction was quenched with 100 µL/well of a stop solution (1 M phosphoric acid). The plates were analyzed using a SpectraMax^®^ 250 spectrophotometer equipped with Softmax Pro 7.1.0 software (Molecular Devices, CA, USA). The endpoint titer was defined as the reciprocal of the minimal dilution whose absorbance (450 nm) was >3 times the background, defined as the average absorbance produced by the wells with a block buffer alone. A statistical analysis was performed using an ordinary one-way ANOVA with Tukey’s multiple comparisons test after verifying equal variances between the treatment groups. As a positive control, we used pooled sera generated in-house from mice that received multiple doses of the DTP. As a negative control, we used pooled sera from mice that were mock-immunized with a dilution buffer (PBS).

### 2.5. Multiplex Immune Assay

The antigens of interest pertussis toxin (PT), pertactin (PRN), filamentous hemagglutinin (FHA), and adenylate cyclase toxin (ACT) were coupled to Magplex-C microspheres according to the manufacturer’s instructions (Luminex Corp., Austin, TX, USA). Next, each antigen-coupled bead was vortexed and sonicated for 10 s, pooled, and combined at a 1:50 dilution in PBN buffer (PBS, 1% BSA, 0.05% sodium azide). Thereafter, 50 µL of bead mixture was added to 50 µL of serum samples previously diluted 1:100 in each well of a black, opaque, 96-well, flat-bottom plate (Greiner Bio-One, Monroe, NC, USA). The plates were incubated for 1 h at room temperature in the dark with shaking at 600 rpm on a tabletop microplate shaker (Thermo Scientific, Waltham, MA, USA). After 1 h, the samples were manually washed by dispensing 200 µL of Luminex wash buffer (PBS, 2% BSA, 0.02% Tween 20, 0.05% azide, pH 7.5) into each well for 2 min with the plates mounted on a 96-well plate magnetic separator. After washing, the samples were incubated with 100 μL of a 1:500 diluted phycoerythrin (PE)-tagged goat anti-mouse IgG-Fc (Southern Biotech, Birmingham, AL, USA) in the dark at RT with shaking at 600 rpm for 30 min. The beads were washed again as described above, and the resultant bead-coupled antigen–antibody complexes were resuspended in 100 µL Luminex wash buffer and incubated for 1 min with shaking (600 rpm) at room temperature in the dark. The measure of antigen-specific—antibody binding in each sample was then measured using a FlexMap 3D instrument (Luminex Corp., Austin, TX, USA) where the results are reported as the Median Fluorescence Intensity (MFI). Statistical differences were measured using a two-way ANOVA with Tukey’s multiple comparisons test.

### 2.6. Antigen–Aluminum Adjuvant Adsorption Assays

To measure the level of adsorption of most of the antigens (i.e., t-NRRV, D, T, Hib, and HepB) to aluminum-salt adjuvants, 1 mL aluminum-salt-adsorbed antigen was centrifuged at 1600× *g* for 5 min in a microcentrifuge tube, 0.9 mL supernatant was removed (0.9 mL represents maximum supernatant volume that was removed without disturbing the pellet), and the pellet was resuspended in 0.9 mL of the same buffer. The supernatant, resuspended pellet, and unfractionated sample were each analyzed using the competitive ELISA and level of antigen adsorption to the adjuvant was expressed as percentage bound antigen, i.e., total antigen in sample (100%)—% unbound antigen in the supernatant. Overall, the total amount of antigen measured (bound + unbound antigen) was similar to the initial amount of antigen added during vaccine preparation. For example, the average mass balance value determined for the formulations described in Section 3.3 was 96 ± 9%.

To measure the level of adsorption of the wP antigen to aluminum-salt adjuvants, we adapted a previously described method for separating inactivated bacteria antigens from aluminum adjuvants via centrifugation through a sucrose cushion [32]. Briefly, 150 µL of the wP antigen containing a vaccine sample was layered over 150 µL of a 60% sucrose cushion in a PCR plate and centrifuged for 1500× *g* for 10 min to separate the non-adsorbed wP antigen from the aluminum-salt adjuvant. After centrifugation, 275 µL of the supernatant (containing non-adsorbed wP) was removed from the pellet fraction (containing alum-adsorbed wP). A 30% sucrose solution was added to the supernatant and pellet fractions to a final volume of 300 µL and gently mixed. The total protein concentration of whole drug product, supernatant, and reconstituted pellet were assayed by a BCA analysis to determine the extent of the wP binding to the aluminum adjuvant [32]. 

### 2.7. Compatibility Studies

The compatibility of AH-adsorbed trivalent NRRV antigens (P[4], P[6], and P[8]) upon simple mixing with aluminum-salt (AH and AP)-adsorbed pentavalent antigen (DTwP-Hib-Hep B) samples was studied by first preparing the two samples at 2× concentrations each of the t-NRRV and a mock pentavalent vaccine formulation at a target pH of 7 in a PBS buffer (0.5 mM phosphate and 150 mM NaCl, pH 7.0). The mock 2× pentavalent vaccine formulation was prepared by combining calculated amounts of stock solutions of the pentavalent antigens followed by the addition of the alum-adjuvants to a final concentration of 80 Lf/mL of D, 40 Lf/mL T, 48 OU/mL wP, 50 µg/mL Hep B, 40 µg/mL Hib, 1.32 mg/mL AP, and 1.0 mg/mL AH under stirring conditions and incubated at 2–8 °C overnight to allow the adsorption of antigens to the adjuvants. Mock 6× t-NRRV vaccine formulations were prepared by combining calculated amounts of stock solution of each NRRV antigen (P[4], P[6], and P[8]) with AH to a final concentration of 360 µg/mL of each NRRV and 2.25 mg/mL of AH under stirring conditions and incubated at 2–8 °C overnight. All three monovalent AH-adsorbed NRRV antigen samples were mixed in equal volumes to prepare a 2× trivalent NRRV vaccine formulation at a concentration of 180 µg/mL of each NRRV and 2.25 mg/mL of AH to a target pH of 7.0.

After preparation of each of the above 2× mock vaccine formulation samples described, 1 mL each were then mixed to prepare 2 mL of 1× hexavalent vaccine (40 Lf/mL of D, 20 Lf/mL T, 24 OU/mL wP, 25 µg/mL Hep B, 20 µg/mL Hib, 60 µg/mL of each NRRV (P[4], P[6], and P[8]), 0.66 mg/mL AP, and 1.625 mg/mL AH at a target pH of 7.0) in stoppered 5 mL glass vials and stored upright at 2–8 °C overnight (i.e., at time zero), followed by the ELISA analysis to measure antibody binding and the degree of adsorption of the three NRRV antigens (P[4], P[6], and P[8]) and the four pentavalent antigens (D, T, Hib, and Hep B). The wP component of the mock pentavalent and mock hexavalent vaccines was evaluated using the mouse immunogenicity assay, and its degree of adjuvant adsorption was measured using the sucrose gradient method described above.

Investigation of the desorption of each NRRV antigen from AH within the mock DTwP combination vaccine was carried out by the addition of 1 mL individual components of DTwP (i.e., to a final concentration of 20 Lf/mL D, 15 Lf/mL T, 24 OU/mL wP, and 0.66 mg/mL AP) to 1 mL monovalent AH-adsorbed NRRV samples (each NRRV at 180 µg/mL and 1.125 mg/mL AH) to prepare 2 mL of combined sample. The sample was stored in 5 mL glass vials in an upright orientation overnight at 4 °C before analysis using the NRRV ELISA to measure percent desorption. Experiments to evaluate mitigation of t-NRRV desorption from adjuvants by using only an AH adjuvant (without AP) were performed as outlined above in the compatibility study, except the mock pentavalent formulation sample was prepared using the AH adjuvant only.

### 2.8. Stability Studies

Real-time (2–8 °C) and accelerated stability studies (15, 25 °C) were carried out using one of the following: (1) mock hexavalent vaccine formulations (after simple mixing of AH-adsorbed t-NRRV and mock pentavalent vaccine containing both AH and AP as described above), (2) AH-only mock hexavalent formulation (after simple mixing AH-adsorbed t-NRRV and mock pentavalent vaccine containing only AH), and (3) control formulations of each without mixing together (i.e., AH-adsorbed t-NRRV alone and mock pentavalent vaccine formulation alone). For these stability studies, 4 mL of each formulation was dispensed in 5 mL sterile glass vials, stoppered with rubber stoppers, and crimped with aluminum seals and stored upright at the above-mentioned temperatures. Stability losses by the competitive ELISA (or mouse immunogenicity for the wP component) and degree of adsorption for each antigen to the aluminum-salt adjuvants were measured as described above. 

### 2.9. Stability Profile of AH-Adsorbed t-NRRV Antigens with Preservatives

The stability of NRRV antigens with eight different individual preservatives was studied by mixing 2× concentrations each of the AH-adsorbed t-NRRV (P[4], P[6], and P[8]) sample with a preservative stock solution to prepare 1× formulation at 0.18 mg/mL total NRRV proteins (0.06 mg/mL of each P[4], P[6] and P[8]), 1.125 mg/mL aluminum, 1× target preservative concentration in 0.5 mM phosphate, 150 mM NaCl, 0.009% polysorbate 80, pH 7.0. The target preservatives concentrations were as follows: (0.25 mM Thimerosal (TH), 72.4 mM 2-Phenoxy ethanol (2-PE), 53.2 mM Phenol (PH), 26.8 mM Chlorobutanol (CB), 27.8 m-cresol (MC), 92.5 mM Benzyl alcohol (BA), 11 mM Methyl paraben, and 0.6 mM propyl paraben (MP + PP)). A volume of 1.4 mL of each multidose formulation was filled in 2 mL glass vials, stoppered, and stored upright at 2–8 °C overnight before incubating at 2–8 °C and 15 °C for up to 12 weeks. The stability profile of each AH-adsorbed NRRV antigen in the presence and absence of these preservatives was measured by relative antibody binding over time using the competitive ELISA as described above.

## 3. Results

### 3.1. Competitive ELISA Development for t-NRRV, D, T, Hib, and Hep B Antigens

**Figure 1:**
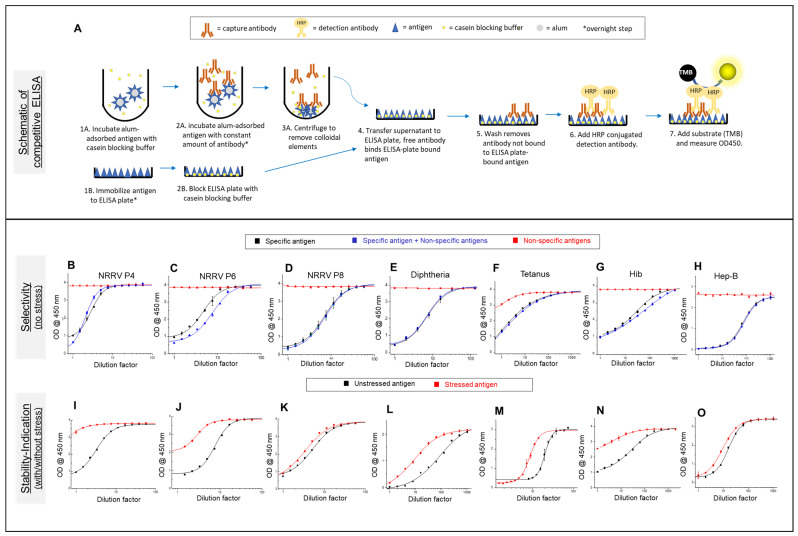
Summary of the competitive ELISA results for assessing the compatibility and stability of aluminum-salt-adjuvanted t-NRRV, D, T, Hib, and Hep B antigens in the presence of one another and the wP antigen. Schematic of the competitive ELISA format (**A**). The selectivity and stability-indication results for NRRV P[4] (**B**,**I**), P[6] (**C**,**J**), P[8] (**D**,**K**), Diphtheria (**E**,**L**), Tetanus (**F**,**M**), Hib (**G**,**N**), and Hep B (**H**,**O**) are shown. See Methods section for description of the samples and stress conditions. Data are presented as the mean ± SD (n = 2–3).

Competitive ELISAs were utilized to assess the compatibility of NRRV antigens (P[4], P[6], and P[8]) with each of the components of the pediatric combination vaccines, in the presence and absence of an aluminum adjuvant (AH and/or AP). The use of the NRRV antigen-specific monoclonal antibodies (Table 1) enables one to monitor the stability profile of conformational or linear epitopes within each antigen while bound to the aluminum-salt (alum) adjuvant. Specifically, previous work has demonstrated the suitability of using NRRV competitive ELISAs for measuring vaccine potency based on correlations between the outcomes of the in vitro ELISAs with in vivo immunization studies in guinea pigs, using a variety of different antigen doses and stressed samples [26]. A schematic of the competitive ELISA format for measuring antibody binding of aluminum-salt-adsorbed NRRV samples is shown in Figure 1A. This assay format was similarly used for the D, T, Hib, and Hep B antigens as described below. The key advantage of measuring antibody binding to adsorbed antigens is there is no requirement to desorb the antigen from the adjuvant (as is required with sandwich ELISA formats), which can lead to different conformational properties than the immunologically relevant adsorbed antigen [26].

First, for each alum-adjuvanted NRRV (P[4], P[6], and P[8]) antigen, the competitive ELISAs were shown to be selective (Figure 1B–D) in the presence of pentavalent antigens (i.e., upon addition of D, T, wP, Hib, and HepB formulated with different aluminum-salt adjuvants, AH + AP) and stability-indicating (Figure 1I–K) by itself (i.e., AH-adsorbed t-NRRV). Selectivity was assessed in the absence of any stress condition by running a known concentration of the three samples: (1) a specific antigen alone (assay standard), (2) alum-adsorbed specific antigens in the presence of the non-specific antigens, (3) alum-adsorbed non-specific antigens minus the specific antigen. These results indicate equivalent antibody binding for samples (1) and (2), while no antibody binding was observed for sample (3). The stability-indicating nature of the assay for each NRRV antigen was tested by measuring the antibody binding of unstressed and heat-stressed (see Methods section) alum-adsorbed samples. The results show a measurable loss in antibody binding in the case of heat stressed vs. unstressed samples of each of the three alum-adjuvanted NRRV antigens.

Similar competitive ELISA method development work was then performed with the D, T, Hep B, and Hib antigens in the presence of one other and the t-NRRV antigens, again in samples with different aluminum-salt adjuvants (AH + AP or AH-only; see Methods). For these four antigens, we did not have access to monoclonal antibodies used in quality control testing labs, hence we purchased antigen-specific antibodies from different sources (Table 1), developed the competitive ELISA format (Figure 1A) for each antigen, and used them to assess the stability profile of the D, T, Hep B, and Hib antigens. It is important to point out, however, that these antibody reagents were not necessarily linked to in vivo performance and were used in this work solely as a “proof-of-concept” for the combination vaccine formulation development work.

For tetanus and diphtheria antigens, we obtained antigen-specific, conformational mAbs from National Institute for Biological Standards and Control (NIBSC), which has reported a sandwich ELISA to monitor D and T antigen concentrations in solution, or upon dissolution of an alum-adjuvant, in combination vaccines [28,29]. For the Hib and Hep B antigens, antibodies were procured from commercial sources (Table 1). The mAb for the Hep B antigen binds a known conformational epitope, while for Hib, antibody binds the linear epitope of the polyribosyl ribitol phosphate (PRP) region of the protein-polysaccharide conjugate antigen. Competitive ELISAs were developed for each alum-adsorbed D, T, Hib, and Hep B antigen and both selectivity (Figure 1E–H) and stability-indicating (Figure 1L–O) properties of the assays were demonstrated using the same criteria described above for the competitive ELISAs with the t-NRRV antigens (see Methods section).

### 3.2. Mouse Immunogenicity Assays and Adjuvant-Adsorption Assay for wP Antigens

**Figure 2:**
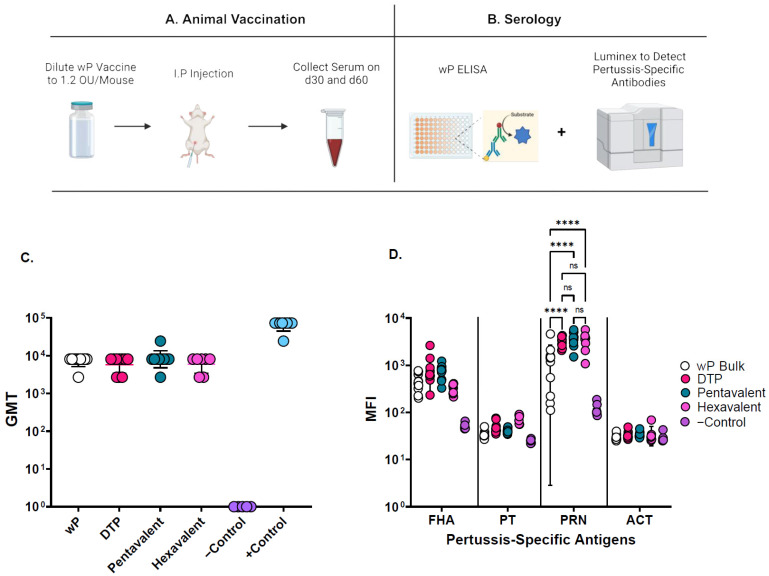
Overview of the mouse immunogenicity assay for assessing the compatibility of inactivated wP antigens in the presence of aluminum-salt-adjuvanted t-NRRV, D, T, Hib, and Hep B antigens. (**A**) Vaccination and serum collection schedule for BALB/c mice, (**B**) whole-cell ELISA for measuring the total antibodies in mice serum, and the multiplex Luminex assay for antigen-specific antibodies for the antigenic components of pertussis. (**C**) The total and (**D**) antigen-specific antibody responses against *B. pertussis* are shown. Error bars in (**C**,**D**) represent geometric standard deviation and standard deviation, respectively. Asterisks and ns indicate either significant differences or no significant differences respectively in antibody responses to pertactin (Tukey’s multiple comparisons test at a *p* value of <0.05).

The mouse immunogenicity assay was used in this work to evaluate the compatibility and stability of inactivated wP antigen in the presence of aluminum-salt-adjuvanted t-NRRV, D, T, Hib, and Hep B antigens. Briefly, a schematic overview of the mouse immunization assay to generate sera for testing is shown in Figure 2A. The whole-cell ELISA involved coating the ELISA plates with *B. pertussis* ATCC 18323 to measure total anti-wP antibodies in serum samples (Figure 2B). While the multiplex Luminex assay was useful for measuring antigen-specific antibodies for commercially available components of pertussis antigens (PRN: pertactin, FHA: filamentous hemagglutinin, PT: pertussis toxin, ACT: Adenylate cyclase toxin) (Figure 2B). Method development, including assessing any changes in the levels of immune responses generated against wP antigens in the various formulations, was carried out using the wP immunogenicity assay. This involved vaccinating BALB/c mice intraperitoneally on day 0 with wP-containing samples and collecting blood on days 30 and 60 (see details in Method Section). The analysis of the serum using both bacterial whole-cell ELISAs and a multiplex immune assay revealed that the monovalent, trivalent, pentavalent, and hexavalent whole-cell pertussis vaccine formulations stimulated high immune responses against *B. pertussis* and other multiplexed vaccine antigens. More specifically, no notable differences in the total and specific anti-pertussis antibodies among the vaccine formulations were observed, although the PRN bulk antibody titers were slightly reduced (Figure 2C,D). The wP assay was selective and showed no notable differences in the readouts of wP alone and when in presence of other hexavalent antigens (D, T, Hib, Hep B, and t-NRRV).

Finally, the adsorption of the wP antigen to the aluminum-salt adjuvants was evaluated by centrifugation through a sucrose cushion (see Methods section and [32]) to determine the percent bound wP antigen. 

### 3.3. Compatibility of Aluminum-Salt-Adjuvanted Formulation of t-NRRV and DTwP-Hib-Hep B When Mixed Together

The most straightforward experiment to initially evaluate the compatibility (at time zero) and stability (over time) was to mix AH-adsorbed t-NRRV antigens with a mock formulation of an aluminum-salt-adjuvanted DTwP-Hib-Hep B mixture. To this end, we prepared a mock formulation of the pentavalent combination vaccine at the lab scale using individual bulks and commercially purchased aluminum-salt adjuvants (see Methods section) at antigen and adjuvant doses in the range reported with commercially available combination vaccines [8,9].

**Figure 3:**
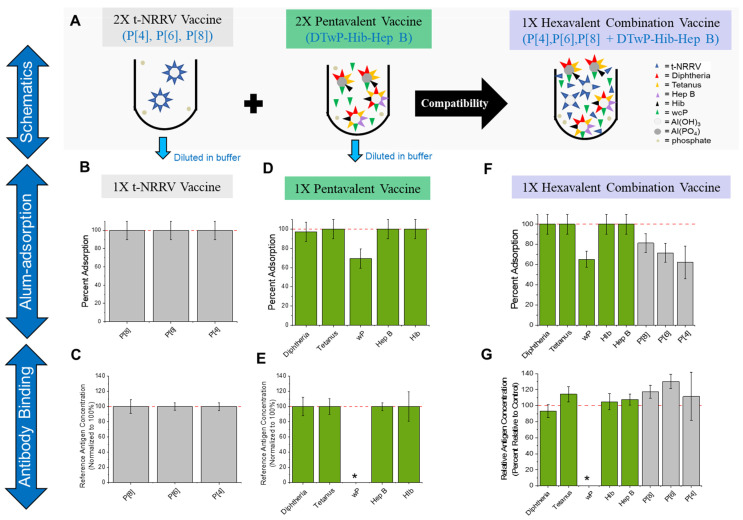
Compatibility of recombinant t-NRRV antigens with pentavalent antigens (D, T, wP, Hib, and Hep B) in hexavalent combination vaccine (t-NRRV + pentavalent) antigens. (**A**) is a schematic representation of adsorption of each antigen in t-NRRV, pentavalent vaccine, and hexavalent vaccine. Alum-adsorption and antibody-binding (relative antigen concentration normalized to 100% for each antigen) results for t-NRRV (**B**,**C**), “default” pentavalent vaccine (**D**,**E**) and hexavalent vaccine (**F**,**G**) are shown. Antibody binding and adsorption for all antigens except wP were measured using the ELISA. For the wP antigen, the adsorption study was performed separately (see Methods), and in vivo mouse immunogenicity results (*) are presented in Section 3.5. Data are presented as the mean ± range (n = 2). When antigen adsorption to alum values were 100%, a range of ±10% was assigned based on the estimated LOQ of the assay.

The initial compatibility study evaluated the simple mixing of AH-adjuvanted t-NRRV with AH + AP-adjuvanted pentavalent antigens (see schematic in Figure 3A). The three NRRV recombinant antigens P[4], P[6], and P[8] were initially completely adsorbed to the AH adjuvant (Figure 3B) and displayed full antibody binding activity (compared to respective bulk standards) (Figure 3C). The pentavalent formulation displayed ~100% alum binding for the D, T, Hib, and Hep B antigens and ~70% for the wP antigen (Figure 3D), and full antibody binding activity (compared to respective bulk standards) (Figure 3E). Upon mixing the pentavalent antigens with the t-NRRV antigens, each of the NRRV antigens became partially desorbed from AH (20–40% desorption), while the percent of pentavalent antigens bound to alum was unaffected (Figure 3F). The in vitro antibody binding results (percent concentration relative to a control) of each of the antigens (NRRV P[4], P[6], and P[8], as well as D, T, Hib, and HepB) showed no notable destabilizing effect (Figure 3G). No differences in the immunogenicity of the wP antigen were observed (shown later in the stability studies section). Overall, however, incompatibility was observed since the observed partial desorption of each of the three recombinant NRRV antigens from the AH adjuvant is anticipated to lower the in vivo immunogenicity readouts after immunization with the three antigens [33] (also see Discussion).

### 3.4. Mitigation of t-NRRV Desorption from AH Adjuvant upon Mixing with the Pentavalent Formulation

**Figure 4:**
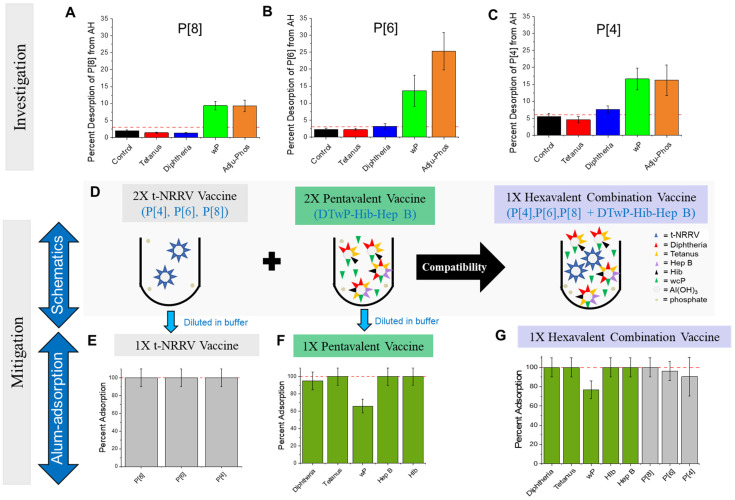
Investigation and mitigation of t-NRRV antigen desorption from aluminum-salt adjuvants when formulated with the components of a mock pentavalent combination vaccine. (**A**–**C**) represent the investigation of NRRV antigen desorption from AH adjuvants for each monovalent adsorbed NRRV antigen by the individual components of a mock DTP vaccine with P[8] (**A**), P[6] (**B**), and P[4] (**C**). (**D**) is schematic representation of adsorption of each antigen, in t-NRRV, the pentavalent vaccine, and the “mitigated” hexavalent AH combination vaccine. (**E**–**G**) represent the percentage adsorption of each antigen when formulated as a (**E**) t-NRRV control (AH adjuvant only), (**F**) mock mitigated pentavalent formulation (AH adjuvant only with D, T, wP, Hib, and Hep B antigens), and mock hexavalent formulation (AH adjuvant only with t-NRRV, D, T, wP, Hib, and Hep B antigens). Adsorption for all the antigens except wP was measured using ELISA. wP adsorption was performed using a sucrose centrifugation assay (see Methods). Data are presented as the mean ± range (n = 2). When antigen adsorption to alum values were 100%, a range of ±10% was assigned based on the estimated LOQ of the assay.

We first explored if simple adjustments to the formulation could help mitigate the desorption of the t-NRRV antigens from the AH adjuvant upon mixing with the pentavalent formulation. For example, based on previous antigen–adjuvant binding studies in our labs, we evaluated if (1) reducing the formulation pH to 6.0, (2) pre-incubating the vaccine antigens with aluminum-salt adjuvants at 4 °C for 5 weeks, and (3) increasing the AH concentration from 1.125 to 1.625 mg/L were helpful; however, only moderate improvements in retaining t-NRRV binding to the AH adjuvant were observed. Next, a more systematic study was performed by combining each AH-adsorbed NRRV antigen individually with components of a trivalent DTwP combination vaccine (D, T, wP antigens and AP adjuvant). As shown in Figure 4A–C, the addition of the wP antigen or the AP adjuvant resulted in notable desorption of each of the three NRRV antigens from AH while the other antigens had minimal to no effect.

Based on these results, we prepared a pentavalent formulation containing only AH (instead of an AH + AP mixture) and then assessed if this change would mitigate the desorption of the three NRRV antigens upon mixing the t-NRRV antigens together with the pentavalent formulation (see schematic in Figure 4D). In this study, the pentavalent and t-NRRV formulations were prepared at the same concentrations as described in the previous compatibility study (Figure 3), except for replacing AP with AH in the pentavalent formulation. By making this change to the pentavalent formulation, no changes were observed in the adjuvant-binding of the three NRRV antigens (~100% bound before and after mixing) as well as for the pentavalent antigens (~100% bound for the D, T, Hib, and Hep B antigens and ~70% bound for the wP antigens (Figure 4E–G). The in vitro antigen binding results (percent concentration relative to a control) of the antigens in AH-bound t-NRRV (alone), AH-adjuvanted formulated pentavalent (D, T, wP, Hib, HepB), and mixture of the two samples were within the expected values and showed no incompatibility upon mixing (± 30%).

### 3.5. Real-Time and Accelerated Stability Studies with Various Combination Formulations (t-NRRV + Pentavalent Antigens in the Presence of Aluminum-Salt Adjuvants)

Based on the compatibility results described above, we evaluated the stability profile of a mock hexavalent formulation containing t-NRRV antigens + pentavalent (D, T, wP, Hib, Hep B) antigens adjuvanted using either a mixed aluminum adjuvant (AH + AP) or AH alone. In addition, these two mock hexavalent combination vaccine formulations were compared to their respective controls, i.e., mock pentavalent formulation (D, T, wP, Hib, and Hep B using mixed aluminum adjuvants, AH + AP) and AH-adsorbed t-NRRV. The samples were stored at 2–8 °C, 15 °C, and 25 °C, and the stability profiles were evaluated by a combination of aluminum-adjuvant binding studies (for each of the eight antigens), antibody binding studies (for each of the antigens except wP), and mouse immunogenicity evaluations (wP component).

First, we evaluated the antigen–adjuvant interactions in the various formulations (using antigen–aluminum adjuvant adsorption assays; see Methods), and similar results were observed during the stability study (over 6 months) as those noted previously in the comparability studies (described above) with ~100% adsorption of t-NRRV antigens in the control and the AH-only hexavalent formulations, but with partial desorption (~25–50%) in the AH + AP-adjuvanted hexavalent formulation. The adsorption profiles of the t-NRRV antigens to the aluminum adjuvants remained unchanged over time in the various formulations 4 °C, 15 °C, and 25 °C (up to 6 months), except for an apparent increase in P[6] adsorption which was likely an artifact due to the aggregation of unbound P[6] over time (i.e., aggregated protein pelleted with the adjuvant during the binding assay (Supplemental Figure S1). Similarly, four of the pentavalent antigens (D, T, Hib, and Hep B) remained ~100% adsorbed and the wP antigen was ~50–80% adsorbed to aluminum adjuvants in all three formulations at 4 °C, 15 °C, and 25 °C (up to 6 months) (Supplemental Figure S2). Overall, the stability results for antigen–adjuvant binding are consistent with the formulation effects on the antigen–adjuvant interactions described above with the compatibility studies (time zero results immediately after mixing together), indicating the antigen–adjuvant interaction effects occur essentially immediately after mixing and do not notably change over time.

**Figure 5:**
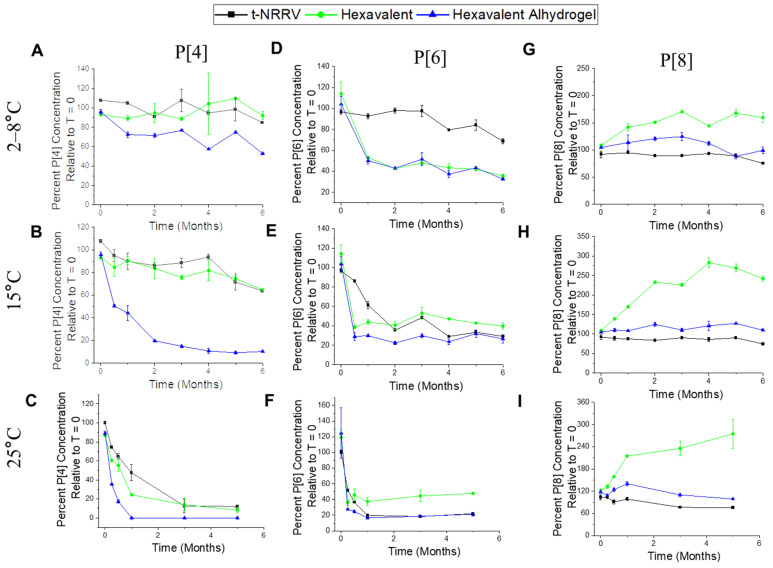
Antigen–antibody binding results (percentage concentration relative to T = 0 using the competitive ELISA) for each of the recombination protein NRRV P[4], P[6], and P[8] antigens formulated as a t-NRRV control (AH adjuvant only), mock hexavalent formulation (AP + AH adjuvants with t-NRRV, D, T, wP, Hib, and Hep B antigens), and mock hexavalent AH formulation (AH adjuvant only with t-NRRV, D, T, wP, Hib, and Hep B antigens) during storage at 2–8 °C, 15 °C, and 25 °C. Antigen–antibody binding for the P[4] antigen at 2–8 °C (**A**), 15 °C (**B**), and 25 °C (**C**) in the three formulations. Antigen–antibody binding for the P[6] antigen at 2–8 °C (**D**), 15 °C (**E**), and 25 °C (**F**) in the three formulations. Antigen–antibody binding for the P[8] antigen at 2–8 °C (**G**), 15 °C (**H**), and 25 °C (**I**) in the three formulations. Samples at 25 °C were assayed more frequently in the first month, and studies were performed up to 5 months (instead of 6 months for samples stored at other temperatures. Data are presented as mean ± SD (n = 4).

In contrast, the stability profiles of the aluminum-salt-adjuvanted t-NRRV antigens as measured by the competitive ELISA (binding to antigen-specific mAbs; see above) displayed changes over time in the three formulations, i.e., the control (AH-adsorbed t-NRRV without pentavalent antigens) and two hexavalent formulations (adjuvanted with either AH + AP or AH-only). For the P[4] antigen, after storage up to 6 months at 4 °C (Figure 5A), minimal losses were observed in the control and the AP + AH hexavalent formulation, while the AH hexavalent formulation demonstrated more losses in antibody binding overtime leading to ~40% loss at 6 months. Under accelerated conditions (15 and 25 °C over 6 months), each of the formulations showed varying degrees of losses in antibody binding, while their above rank ordering remained unchanged as shown in Figure 5B,C, respectively. For the other two NRRV antigens, the P[6] antigen was the least stable across the different storage temperatures, with the control formulation displaying an improved stability profile compared to either of the two hexavalent formulations (Figure 5D–F), a result showing destabilization of the P[6] antigen by the components of the pentavalent formulation. In contrast, the P[8] antigen displayed the best stability profile of the three NRRV antigens, with minimal losses in antibody binding observed in the three formulations at 2–8, 15, and 25 °C up to 6 months, respectively (Figure 5G–I), a result showing no notable destabilization of the P[8] antigen by the components of the pentavalent formulation. Interestingly, the P[8] antigen in the AH + AP-adjuvanted hexavalent formulation demonstrated an overestimation of stability at all the studied temperatures (2–8, 15, and 25 °C). This result is likely due to a combined effect of this formulation containing partially desorbed P[8] antigens (~25–50% desorbed), and the previously reported effect of TH (present from the wP bulk; see Discussion) leading to an increase in binding to the more accessible linear epitope on P[8] recognized by the 7H7 capture antibody.

**Figure 6:**
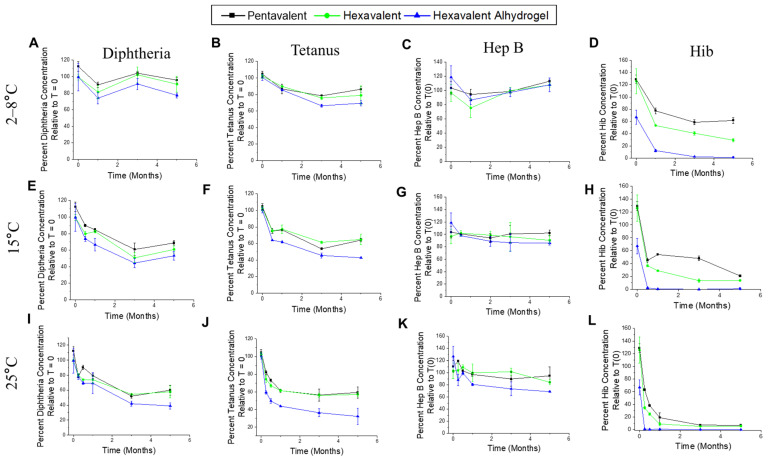
Antigen–antibody binding results (percentage concentration relative to T = 0 using the competitive ELISA) for each of four antigens (D, T, Hep B, and Hib) formulated as a mock pentavalent control (AP + AH adjuvants with D, T, wP, Hib, and Hep B antigens), mock hexavalent formulation (AP + AH adjuvants with t-NRRV, D, T, wP, Hib, and Hep B antigens), and mock hexavalent AH formulation (AH adjuvant only with t-NRRV, D, T, wP, Hib, and Hep B antigens) during storage at 2–8 °C, 15 °C, and 25 °C for 5 months. Antigen–antibody binding at 2–8 °C for diphtheria (**A**), tetanus (**B**), Hep B (**C**), and Hib (**D**). Antigen–antibody binding at 15 °C for diphtheria (**E**), tetanus (**F**), Hep B (**G**), and Hib (**H**). Antigen–antibody binding at 25 °C for diphtheria (**I**), tetanus (**J**), Hep B (**K**), and Hib (**L**). Data are presented as mean ± SD (n = 4).

We then evaluated the stability profile of the aluminum-salt-adjuvanted pentavalent antigens in the same two mock hexavalent formulations (adjuvanted with either AH + AP or AH-only), but in this case, compared to a different control (pentavalent formulation in AH + AP without the t-NRRV antigens). For four of the pentavalent antigens (D, T, Hib, and Hep B), the stability profile was monitored by the competitive ELISAs (binding to antigen-specific mAbs; see above) in the various formulations at 2–8 °C, 15 °C, and 25 °C (up to 6 months). At 4 °C, no notable losses in antibody binding were observed for three of the pentavalent antigens (D, T, Hep B; Figure 6A,B and C, respectively), and no differences were observed in the three formulations. At accelerated temperatures (15, 25 °C), expected losses in antibody binding values were seen, again with no differences noted between the formulations for three of the pentavalent antigens (D, T, Hep B; Figure 6E–G,I–K). For the Hib antigen, instability was observed in all the formulations at both 2–8 °C (Figure 6D) and accelerated storage conditions (15 °C and 25 °C) (Figure 6H,L), with the most destabilization seen in the AH-only hexavalent formulation. This loss of antibody binding of the Hib antigen over time is likely due to the known interaction of the negatively charged Hib antigen with the positively charged AH adjuvant, leading to steric hinderances as well as chemical instabilities [34,35]. Overall, these results indicate the t-NRRV antigens have no effect on the stability of the four pentavalent antigens.

**Figure 7:**
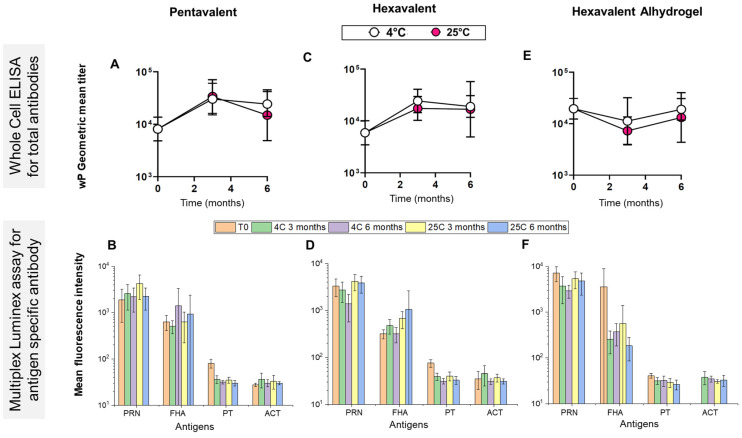
Stability of the whole-cell pertussis antigen (wP) in three different formulations as measured by the mouse immunogenicity assay. Samples include a mock pentavalent formulation control (D, T, wP, Hib, and Hep B formulated with AH + AP adjuvants), mock hexavalent formulation (t-NRRV, D, T, wP, Hib, and Hep B formulated with AP + AH adjuvants) or mock hexavalent-Alhydrogel formulation (t-NRRV, D, T, wP, Hib, and Hep B formulated with AH adjuvant only). (**A**,**C**,**E**) Geometric mean titers obtained using whole-cell ELISA for the wP antigen in the three formulations stored at 2–8 °C and 25 °C at different timepoints, with error bars representing geometric standard deviation (**B**,**D**,**F**). Mean fluorescence intensity obtained using the multiplex Luminex assay for different components of the pertussis antigen (PRN, FHA, PT, and ACT) for the wP antigen in the three formulations stored at 2–8 °C and 25 °C at different timepoints with error bars representing the standard deviation of the mean fluorescence intensity. Data are presented as the mean ± SD (n = 8–10).

Finally, the stability profile of the wP component of the same three mock combination vaccine formulations was measured using the mouse immunogenicity assay for the samples stored at 4 °C and 25 °C over 6 months. Mouse sera were analyzed for both the ELISA (geometric mean titers) and multiplex Luminex assay (mean fluorescence intensities for the PRN, FHA, PT, and ACT components of pertussis antigens). The wP component in the pentavalent control (Figure 7A,B), AH + AP-adjuvanted hexavalent (Figure 7C,D), and AH-only adjuvanted hexavalent (Figure 7E,F) formulations were shown to be stable when stored up to 6 months at 4 °C and 25 °C. No notable changes in the geometric mean titers (ELISA) or mean fluorescence intensities (Luminex) were observed in for the wP component of the three different formulations. These results demonstrate the t-NRRV antigens have no effect on the stability of the wP antigen.

### 3.6. Effect of Preservatives on AH-Adsorbed t-NRRV as Measured by Competitive ELISA

Previous work in our lab has demonstrated that some of the NRRV antigens are destabilized by TH and 2-PE, the two most commonly used preservatives in multidose pediatric combination vaccines [36,37]. Therefore, adding the t-NRRV antigens into a multidose combination vaccine formulation will require the identification of alternative preservatives and/or replacement of TH as the inactivation agent for preparation of the inactivated wP component (see Discussion). As an initial study to assess the feasibility of the former approach, we evaluated the stability profile of AH-adsorbed t-NRRV (P[4], P[6], and P[8]) in the presence of eight different preservatives found in parenterally administered vaccines and drugs (see Discussion). We used an experimental design previously reported for the monovalent AH-adsorbed P[4] antigen [37], and we measured stability by competitive ELISAs for each of the three NRRV antigens (as described above).

**Figure 8:**
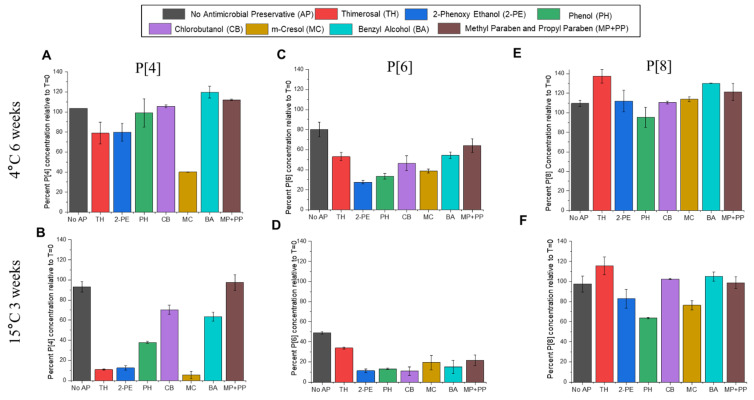
Stability profile of the AH-adsorbed recombinant protein t-NRRV antigens (P[8], P[6], and P[4]) in the presence of the different preservatives as measured by the competitive ELISA. The relative antigen binding of each antigen to an antigen-specific mAb is shown as a percentage concentration relative to time zero values after storage at 2–8 °C for 6 weeks and 15 °C for 3 weeks. Percent antigen–antibody binding for (**A**,**B**) the P[4] antigen, (**C**,**D**) the P[6] antigen, and (**E**,**F**) the P[8] antigen in the presence and absence of the indicated preservatives. Data are presented as the mean ± SD (n = 4).

The loss of antibody binding during storage at 2–8 °C for 6 weeks and 15 °C for 3 weeks for each of the three NRRV antigens (formulated as AH-adsorbed t-NRRV mixture) is shown for the P[4] (Figure 8A,B), P[6] (Figure 8C,D), and P[8] (Figure 8E,F) antigens, respectively. The three NRRV antigens display different relative stability profiles (compared to each other) with the P[8] antigen being the most stable, the P[6] antigen being the least stable, and the P[4] antigen showing intermediate stability. This rank ordering of the antigen stability profiles was consistent in the absence and presence of the various preservatives over the time course of this study. The P[8] antigen showed minimal destabilization without preservatives and only relatively minor effects due to the addition of the eight different preservatives. In contrast, the P[6] antigen was notably the least stable in the absence of the preservatives and was further destabilized by the addition of the preservatives. The P[4] antigen showed the most differentiation between the destabilizing effects of the various preservatives. For example, thimerosal, 2-PE, and m-cresol had the most destabilizing effect on the P[4] antigen, while chlorobutanol, benzyl alcohol, and the parabens (methyl and propyl paraben) were the least destabilizing for the AH-adsorbed P[4] antigen.

## 4. Discussion

The addition of a new recombinant antigen into established pediatric combination vaccines offers many public health enhancements including improved coverage and compliance with vaccination schedules as well as lower costs with fewer vaccine doses and immunization visits (see Introduction; [38]). From a formulation development perspective, previous reports have largely focused on the introduction of new antigens into the pediatric combination vaccines containing acellular pertussis (aP) antigens (i.e., D, T, aP, Hib and Hep B) [1]. The implementation of isolated pertussis protein antigens (as a replacement for inactivated wP bacterium) results in improved storage stability, likely due to fewer destabilizing interactions between the antigens (and between their formulation components such as adjuvants and preservatives). Moreover, fewer interferences with in vitro analytical methods enable more successful formulation development work. For example, both physicochemical and in vitro potency assays for aP antigens within combination vaccines have been reported [39].

Over the past decades, there has been a particular focus on adding trivalent inactivated polio antigens (t-IPV containing IPV Types 1,2,3) to the pediatric combination vaccines to produce a hexavalent vaccine using aP antigens (i.e., D, T, aP, Hib, Hep B, and IPV) [2]. At the same time, efforts to add the inactivated polio vaccine (t-IPV antigens) to the pentavalent vaccine containing wP antigens (i.e., D, T, wP, Hib, Hep B, and IPV), which is highly desired for applications in LMICs, have encountered numerous technical challenges. For example, it is difficult to make such a combination vaccine as the preservative Thimerosal (TH), used during the wP bulk inactivation process (see Introduction), is destabilizing to IPV antigens [5]. This instability requires either the identification of stabilizers to protect the three IPV antigens and/or the development of a new manufacturing process to produce inactivated wP (see below). The former approach was studied by Kraan et al. (2016) and was not successful even when lyophilized t-IPV was reconstituted with a liquid pentavalent vaccine (with wP antigen and TH) and then promptly administered [40]. Other technical and practical hurdles include (1) instability of the Hib antigen in the presence of aluminum hydroxide adjuvants requiring the use of aluminum phosphate-based adjuvants, (2) limited commercial access to each of the antigens, and (3) the substitution of t-IPV (Salk) antigens with Sabin t-IPV antigens [5,7].

As summarized in the introduction, traditional wP bulks contain TH, which is added in the process together with heat for the inactivation of wP bacteria [41]. Since TH is known to negatively affect the antigenicity and immunogenicity of IPV antigens [42], the production of TH-free wP bulks has been a focus of many DCVMs in order to add IPV antigens to the pentavalent pediatric vaccine [43]. Although there are currently no WHO-prequalified hexavalent vaccines containing both wP and t-IPV available for global use, a few locally approved hexavalent vaccines (i.e., DTwP-Hib-Hep B-IPV) prepared by Indian manufacturers are available, namely, EasySix^TM^ (Panacea Biotec (New Delhi, India)), Shan6^®^ (Sanofi Healthcare), and HEXASIIL^®^ (Serum Institute of India Pvt. Ltd. (New Delhi, India)). These vaccines contain 2-phenoxy ethanol (2-PE) as a preservative and utilize formaldehyde and heat as the wP bulk-inactivating agent [44,45,46,47]. Since developing countries vaccine manufacturers (DCVMs) are actively pursuing to replace TH-inactivated wP bulks with TH-free wP antigen bulks, more hexavalent vaccine candidates containing both wP and IPV in combination are now in the clinical development pipeline (e.g., from LG Life Sciences (Seoul, Republic of Korea) and Biologicals E. Limited (Telangana, India) [45,46,48,49,50]).

Based on the incompatibility experienced with t-IPV antigens added to pentavalent combination vaccine candidates containing inactivated wP antigen (i.e., DTwP-Hib-Hep B), the focus of this work was to assess the types of analytical and formulation challenges that will be encountered when adding any new recombinant protein antigen to the components of pentavalent pediatric combination vaccines used in LMICs. Potential incompatibilities in the formulation could be due to the nature of the antigens, adjuvants, and/or preservatives. To this end, we examined three recombinant protein antigens (t-NRRV) adsorbed to an aluminum hydroxide adjuvant, a formulation which was tested in both early- and late-stage human clinical trials (see introduction) and whose target product profile required eventual addition to the pediatric combination vaccines [19,46,51].

### 4.1. Analytical Challenges and Future Work

It is estimated that 70% of the manufacturing time required to produce combination vaccines containing DTwP can be attributed to the running of QC assays. [52]. Traditional QC tests for D and T antigens formulated with aluminum-salt adjuvants are based on mouse immunogenicity or toxin challenge model testing [53]. For the wP antigen, the QC potency assay is an intracerebral mouse protection assay called the Kendrick test [54]. These animal-based potency assays are labor intensive, time-consuming, expensive, and variable in terms of their biological readouts [55]. Therefore, there is growing interest to replace, reduce, and refine (referred to as the 3Rs principles) the use of animals by developing alternative approaches to potency assays, including in vitro immunological assays, that correlate well with the animal-based QC assays [55]. Some examples of animal-based potency assays that have been replaced by immunochemical binding assays for human vaccines include hepatitis A and B, inactivated polio virus, and human papillomavirus [55]. Similar in vitro potency ELISAs are in development to replace the use of animals for tetanus- and diphtheria-containing vaccines. For wP, alternative animal models are being evaluated including respiratory challenge assays, nitric oxide induction assays, and serological assays to replace the Kendrick assay [28,56].

In this work, for the aluminum-salt-adjuvanted D, T, Hib, and Hep B antigens, we developed in-house surrogate antigen–antibody binding assays to identify incompatibilities upon adding t-NRRV antigens. Our competitive ELISAs were developed using commercially available reagents since we did not have access to monoclonal antibodies used in QC testing labs. Since these antibody reagents are not necessarily linked to in vivo performance, they were used solely as a “proof-of-concept” for the combination vaccine formulation development work. Moving forward, it would be helpful to work with antibody suppliers/manufacturers to procure monoclonal antibodies specific to conformational epitopes on the antigen surface to better understand the stability trends. ELISAs can be very sensitive to subtle changes in the antigen structure which might not necessarily be a true representation of in vivo immunogenicity profiles [57]. Thus, as part of future work, assessing animal immunogenicity for each of the antigens in optimized multidose, combination vaccine formulations will give a better picture of the candidate’s overall stability and immunogenicity profiles. This staged approach to multidose, combination vaccine formulation development work (i.e., initially assess compatibility using ELISAs followed by down-selection of candidate formulations for subsequent animal immunogenicity testing) addresses the practical limitations of performing large numbers of in vivo animal experiments.

In this work, for the wP antigen, we immunized mice intraperitoneally with wP containing vaccines, collected blood, and measured immune responses using bacterial whole-cell ELISA and the multiplex Luminex immune assay (see Methods). Whole-cell ELISA was used for measuring the total antibodies in mice serum, and the multiplex assay was used for measuring antigen-specific antibody for antigenic components of pertussis. Both these assays are “proof-of-concept” assays to measure mouse immunogenicity and will require additional work to establish stability indication (with unstressed vs. stressed samples) and making correlations with the Kendrick assay in the future. For analysis of the recombinant protein NRRV antigens (P[4], P[6], and P[8]), we anticipated that many of the physicochemical analytical tools previously developed to characterize NRRV antigens [58] would be difficult to implement in the presence of the mock hexavalent formulation containing the wP antigen, aluminum-salt adjuvants, and preservatives. Thus, we focused on the use of in vitro antigen–antibody binding assays for t-NRRV antigens (i.e., a competitive ELISA with antigen-specific mAbs; [26,27]) to assess their compatibility within combination vaccines. The suitability of these reagents for use in an in vitro potency assay for t-NRRV antigens and the correlation of results with the outcomes of in vivo immunization studies in guinea pigs has been established [26].

In terms of future work, further development of physicochemical assays to assess the structural integrity of recombinant protein antigens added to a combination vaccine are needed, especially in the context of wP antigens. In the case of combination vaccines containing aP antigens, there have been several promising reports of introducing new physicochemical assays to assess antigen stability in the context of combination vaccines. For example, a recent paper by Duprez et al. has evaluated the structure and compositional analysis of three different Tdap vaccine formulations (Tetanus, diphtheria, and acellular pertussis antigens) containing an aluminum hydroxide adjuvant and a genetically detoxified pertussis toxin (gdPT) using a variety of biophysical characterization tools. The authors demonstrated that the different Tdap formulations were similar in terms of their thermal stability (using nano-differential scanning fluorimetry, DSF), size distribution (laser diffraction, LD), and conformational integrity (overall secondary structures by Fourier transform infrared spectroscopy, FTIR) [59]. In-process characterization of the same combination Tdap vaccine antigens has been performed using in-line particle sizing (FBRM^®^) and IR (ReactIR) process analytical technology (PAT) tools [59]. In a related study, Kalbfleisch et al. utilized several biophysical analytical tools, namely, LD, FTIR, and intrinsic fluorescence spectroscopy (IF), to examine the structural integrity of vaccine antigens adsorbed to an aluminum phosphate adjuvant in commercial acellular pertussis (aP)-containing pediatric quadrivalent and pentavalent vaccines [60].

### 4.2. Formulation Challenges and Future Work

Previous vaccine formulation studies from our labs demonstrated that each of the three recombinant NRRV protein antigens (P[4], P[6], P[8]), when formulated with aluminum-salt adjuvants, are sensitive to common formulation components including a phosphate buffer (leading to desorption from aluminum hydroxide adjuvant, AH), and preservatives such as TH and 2-PE (leading to structural alterations and loss of in vitro potency) [36,37,42]. These NRRV antigen degradation pathways were anticipated to be affected by the addition of the AH-adjuvanted t-NRRV antigens into a mock formulation of the pentavalent combination vaccine (D, T, wP, Hib, and Hep B containing TH preservatives and AH and AP aluminum-salt adjuvants). We identified three observations as “lessons learned” for such multidose, combination vaccine formulation development work including (1) antigen instability due to the presence of other antigens, (2) antigen desorption from aluminum-salt adjuvants, and (3) antigen sensitivity toward preservatives used in multidose formulations.

In this work, one key observation was the inherent instability of the NRRV protein antigens in the presence of the components of the pediatric combination pentavalent vaccine. For example, we observed an accelerated loss in antibody binding for the NRRV P[6] antigen during the real-time (2–8 °C) stability study in the presence of pentavalent antigens and their formulation components. As part of future work, it will be necessary to evaluate the effect of individual pentavalent antigens in terms of NRRV P[6] storage stability. In addition, the wP and AP components of the pentavalent vaccine were shown to desorb the NRRV antigens from the AH adjuvant. Although NRRV antigens adsorbed to AH have been shown to be more immunogenic vs. NRRV antigens without AH in mouse studies [33], there are no studies examining injecting unbound NRRV antigens co-administered with an AH aluminum adjuvant (i.e., when formulated with a phosphate buffer which has been shown to desorb NRRV antigens from AH). Therefore, future work should evaluate if NRRV antigens bound vs. unbound to AH have similar immune responses in animal studies.

A second key observation from this work was that mitigating the desorption of NRRV antigens from the aluminum adjuvant (in the presence of the components of the pediatric combination pentavalent vaccine) led to destabilization of some of the other antigens. For example, while preparing a t-NRRV + pentavalent vaccine formulation using an AH adjuvant only (no AP adjuvant) mitigated the desorption of the NRRV antigens from alum, this formulation caused higher instability of the Hib antigen under real-time (2–8 °C) storage conditions and rapid destabilization under accelerated temperatures (15 and 25 °C). Catalytic depolymerization of phosphodiester bonds of PRP in Hib in the presence of an aluminum hydroxide adjuvant has been reported to cause hydrolysis of the PRP polymer into smaller fragments and release of PRP oligomers from the conjugate [39]. The PRP of Hib is the major virulence factor of the organism and is composed of equimolar proportions of D-ribose, ribitol, and phosphate [61] and thus is negatively charged and can strongly interact with the positively charged AH. This leads to ligand exchange and instability of Hib-PRP when adsorbed to AH. The elevated microenvironment pH on the surface of the aluminum adjuvant can also account for enhanced hydrolysis of the phosphodiester bond of PRP as hydrolysis rates increase under alkaline conditions [62].

One option to address this known instability is to formulate the Hib antigen as a separate, lyophilized component to mitigate its destabilization and reconstituting it with other antigens before administration, using a similar strategy as in Pentacel^®^ [63]. Pentacel^®^ contains a liquid DTaP-IPV component and a vial of lyophilized ActHIB^®^. It requires gently shaking the vial of a DTaP-IPV component, withdrawing the entire liquid content, and injecting it into the vial of the lyophilized ActHIB^®^ vaccine component, followed by gently swirling the vial containing Pentacel^®^ until a cloudy, uniform, white-to-off-white (yellow tinge) suspension results; then, it is ready for administration [63]. Interestingly, it has been reported that the Eupenta^TM^ (LG Chem Ltd., Seoul, Korea) vaccine demonstrates sufficient stability in presence of AH. Eupenta^TM^ is a 2016 WHO-prequalified Hib-containing pentavalent vaccine with ~0.4 mg aluminum in the form of AH per 0.5 mL dose [64]. Eupenta^TM^ contains a phosphate buffer which is known to interact with an AH adjuvant and alter its surface charge (either by decreasing the net positive charge or changing to a net negative charge) [65]. Although speculated, it is possible that the phosphate buffer in this formulation may alter the interactions between Hib and AH.

Finally, a third key observation from this work is that the addition of new antigens to the pentavalent pediatric combination vaccine requires an assessment of preservative compatibility, present due to the multidose format of such presentations. The t-NRRV antigens also have been reported to be sensitive to preservatives [42], as has been described in the current study (Figure 8). Since preservatives will also be required for developing a multidose combination vaccine to further ease cost [25], the final objective of this study was to assess the structural stability of three different NRRV antigens in the presence of eight common preservatives (namely, thimerosal, 2-phenoxy ethanol, phenol, chlorobutanol, m-cresol, benzyl alcohol, methyl, and propyl paraben) to determine top alternative preservatives (and/or their combinations), instead of TH, for the development of a possible multidose combination vaccine. Most of the preservatives were used at their highest in-use concentration [66] except for methyl paraben and propyl paraben which were used at their maximum solubility levels.

Our results showed different relative stabilities of the three NRRV antigens in decreasing order, P[8] > P[4] > P[6], both in the presence and absence of preservatives. In addition, TH and 2-PE, the most commonly used preservatives in combination vaccines, cannot be used due to their high destabilizing effects on all three NRRV antigens during storage stability as measured using antigen–antibody binding competitive ELISAs (see results section). An earlier study from our lab demonstrated the deleterious effects of TH and 2-PE on storage stability of all three NRRV antigens using a variety of analytical methods [36]. Additional studies with the P[4] NRRV antigen demonstrated the mechanism of TH-induced structural destabilization of the antigen via mass spectrometry and hydrogen exchange-mass spectrometry (HX-MS) studies [37,42]. In this work, we identified chlorobutanol, benzyl alcohol and the parabens (methyl and propyl paraben) as the least destabilizing preservative options for preparing a multidose formulation of the aluminum-adjuvanted t-NRRV antigens. Although a longer-term goal, assessing the compatibility of the alternative preservatives with the pentavalent bulk antigens (e.g., wP, D, T, Hib, and Hep B) would provide the baseline data to assess the potential to design new multidose formulations to further ease costs and improve stability.

Finally, future work that screens various types and categories of excipients to further stabilize the NRRV and pentavalent antigens, while mitigating antigen desorption from the alum adjuvant, could also play a helpful role in developing stabilized combination vaccine formulations. This would especially be useful to stabilize the most susceptible antigens in the presence of preservatives for the development of a possible multidose vaccine. To this end, performing temperature excursion stability studies (i.e., short-term exposure to elevated temperatures of up to 50 °C) with optimized multidose formulations would facilitate wider distribution of combination vaccines within LMICs if limited cold chain infrastructures are encountered.

## 5. Conclusions

In this study, we report a case-study evaluating the feasibility of introducing a recombinant subunit rotavirus vaccine candidate (consisting of P[4], P[6], and P[8] antigens formulated with AH aluminum-salt adjuvant) to a mock multidose formulation of a pediatric pentavalent combination vaccine used in LMICs (consisting of DTwP-Hep B-Hib antigens formulated with a mixture of AH and AP aluminum-salt adjuvants and the preservative TH). A series of selective and stability-indicating analytical assays including competitive ELISAs (for t-NRRV, D, T, Hib, and Hep B) and mouse immunogenicity assays (for wP) were developed/set-up and utilized to assess the compatibility and stability of the antigens. The key issues identified for the addition of the candidate recombinant t-NRRV antigens to a mock formulation of a pediatric pentavalent combination vaccine (D-T-wP-Hib-HepB) included sensitivity to specific components of the complex formulation, including the wP antigen and the TH preservative (leading to instability) as well as the AP adjuvant (leading to antigen desorption). We also evaluated a series of alternative preservatives to replace TH as part of future multidose formulation work and identified chlorobutanol, benzyl alcohol, and parabens (methyl and propyl paraben) as preferred options for future multidose formulation development efforts.

For the pentavalent antigens, we did not observe any notable instabilities upon the addition of t-NRRV antigens, with the exception of an AH-only adjuvanted formulation which displayed the known AH-induced instability of the Hib antigen (see text). It is important to note we did not have access to QC-related antibody reagents for the D, T, Hib, and Hep B assays, so we obtained antigen-specific antibodies from different commercial sources. For the wP antigen, we performed a mouse immunogenicity assay and not the QC animal potency test (Kendrick assay). Thus, our results are not linked to in vivo performance and should be viewed solely as “proof-of-concept” data for performing formulation development experiments to assess the feasibility of developing multidose formulations for new recombinant antigens being added to existing pediatric combination vaccines targeted for use in LMICs and thus containing the wP antigen, aluminum-salt adjuvants, and preservatives.

## Figures and Tables

**Table 1:** Overview of the key assays and antibody reagents used in this study to analyze the compatibility and stability of t-NRRV and pentavalent antigens in mock combination formulations containing aluminum-salt adjuvants.

	Aluminum-Salt-Adjuvanted Antigen	Assay	Capture Antibody	Capture Ab Binds	Reference
NRRV antigens	NRRV P[8]	Competition ELISA	mAb 7H7 (Precision Antibody)	Linear epitope of NRRV P[8]	[26,27]
	NRRV P[6]		mAb 3G11 (Precision Antibody)	Linear epitope of NRRV P[6]	
	NRRV P[4]		mAb 13A1 (Precision Antibody)	Conformational epitope NRRV P[4]	
Pentavalent antigens	Diphtheria (D)		mAb 10/130 (NIBSC)	Conformational epitopes on diphtheria toxoid	[28,29]
	Tetanus (T)		mAb NIBSC 10/134 (NIBSC)	Conformational epitopes on tetanus toxin	
	PRP-T conjugate (Hib)		HIB12-S (Alpha Diagnostics)	Hib PRP region	[30]
	Hepatitis B surface antigen (HepB)		mAb 4940-1404 surface sntigen (Bio-Rad)	Conformational epitopes on Hep B surface antigen	[31]
	Whole-cell inactivated pertussis (wP)	Mouse Immunogenicity	N/A	N/A	N/A

N/A—not applicable.

## Data Availability

The datasets presented in the current study are available in the KU ScholarWorks repository at DOI: https://doi.org/10.17161/1808.35079. The data are also available from the corresponding authors.

## Supplementary Materials

The following supporting information can be downloaded at: https://www.mdpi.com/article/10.3390/vaccines12060609/s1, Figure S1: Percent adsorption binding results for each of the recombinant protein NRRV P[4], P[6], and P[8] antigens formulated as a t-NRRV control (AH adjuvant only), mock hexavalent formulation (AP + AH adjuvants with t-NRRV, D, T, wP, Hib, and Hep B antigens), and mock hexavalent AH formulation (AH adjuvant only with t-NRRV, D, T, wP, Hib, and Hep B antigens) during storage at 2–8 °C, 15 °C, and 25 °C; Figure S2: Percent adsorption binding results for each of the five pentavalent antigens (D, T, wP, Hep B, Hib) formulated as a mock pentavalent control (AP + AH adjuvants with D, T, wP, Hib, and Hep B antigens), mock hexavalent formulation (AP + AH adjuvants with t-NRRV, D, T, wP, Hib, and Hep B antigens), and mock hexavalent AH formulation (AH adjuvant only with t-NRRV, D, T, wP, Hib, and Hep B antigens) during storage at 2–8 °C, 15 °C, and 25 °C for 5 months.

## References

[1] Skibinski D.A., Baudner B.C., Singh M., O’Hagan D.T. (2011). Combination Vaccines. J. Glob. Infect. Dis..

[2] Obando-Pacheco P., Rivero-Calle I., Gómez-Rial J., Sánchez C.R.T., Martinón-Torres F. (2018). New Perspectives for Hexavalent Vaccines. Vaccine.

[3] Ma J., Li Z., Sun Y., Liu Z., Dang Y., Huang Y. (2022). Improving Innovation and Access to Combination Vaccines for Childhood Immunization in China. Int. J. Environ. Res. Public Health.

[4] Shashidhar A. (2017). Hexavalent Vaccinations: The Future of Routine Immunization?. Indian. Pediatr..

[5] Mahmood K., Pelkowski S., Atherly D., Sitrin R., Donnelly J.J. (2013). Hexavalent Ipv-Based Combination Vaccines for Public-Sector Markets of Low-Resource Countries. Hum. Vaccin. Immunother..

[6] WHO Thiomersal. https://www.who.int/teams/health-product-policy-and-standards/standards-and-specifications/vaccines-quality/thiomersal.

[7] Kumar P., Bird C., Holland D., Joshi S.B., Volkin D.B. (2022). Current and Next-Generation Formulation Strategies for Inactivated Polio Vaccines to Lower Costs, Increase Coverage, and Facilitate Polio Eradication. Hum. Vaccines Immunother..

[8] WHO List of Prequalified Vaccines. https://extranet.who.int/pqweb/vaccines/list-prequalified-vaccines.

[9] US-FDA Vaccines. https://www.fda.gov/vaccines-blood-biologics/vaccines.

[10] Burnett E., Parashar U., Tate J. (2018). Rotavirus Vaccines: Effectiveness, Safety, and Future Directions. Paediatr. Drugs.

[11] Kirkwood C.D., Ma L.-F., Carey M.E., Steele A.D. (2019). The Rotavirus Vaccine Development Pipeline. Vaccine.

[12] Kumar P., Pullagurla S.R., Patel A., Shukla R.S., Bird C., Kumru O.S., Hamidi A., Hoeksema F., Yallop C., Bines J.E. (2021). Effect of Formulation Variables on the Stability of a Live, Rotavirus (Rv3-Bb) Vaccine Candidate Using in Vitro Gastric Digestion Models to Mimic Oral Delivery. J. Pharm. Sci..

[13] Magwira C.A., Taylor M.B. (2018). Composition of Gut Microbiota and Its Influence on the Immunogenicity of Oral Rotavirus Vaccines. Vaccine.

[14] Harris V.C., Armah S.G., Fuentes K.E., Korpela U., Parashar J.C., Victor J., Tate C., de Weerth C., Giaquinto W.J., Wiersinga K.D. (2017). Significant Correlation between the Infant Gut Microbiome and Rotavirus Vaccine Response in Rural Ghana. J. Infect. Dis..

[15] Parker E.P., Praharaj I., Zekavati A., Lazarus R.P., Giri S., Operario D.J., Liu J., Houpt E., Iturriza-Gómara M., Kampmann B. (2018). Influence of the Intestinal Microbiota on the Immunogenicity of Oral Rotavirus Vaccine Given to Infants in South India. Vaccine.

[16] Lazarus R.P., John J., Shanmugasundaram E., Rajan A.K., Thiagarajan S., Giri S., Babji S., Sarkar R., Kaliappan P.S., Venugopal S. (2018). The Effect of Probiotics and Zinc Supplementation on the Immune Response to Oral Rotavirus Vaccine: A Randomized, Factorial Design, Placebo-Controlled Study among Indian Infants. Vaccine.

[17] Jiang B., Gentsch J.R., Glass R.I. (2018). Inactivated Rotavirus Vaccines: A Priority for Accelerated Vaccine Development. Vaccine.

[18] O’Ryan M., Lopman B.A. (2017). Lopman. Parenteral Protein-Based Rotavirus Vaccine. Lancet Infect. Dis..

[19] Debellut F., Pecenka C., Hausdorff W.P., Clark A. (2022). Potential Impact and Cost-Effectiveness of Injectable Next-Generation Rotavirus Vaccines in 137 Lmics: A Modelling Study. Hum. Vaccin. Immunother..

[20] Wen X., Cao D., Jones R.W., Hoshino Y., Yuan L. (2015). Tandem Truncated Rotavirus Vp8* Subunit Protein with T Cell Epitope as Non-Replicating Parenteral Vaccine Is Highly Immunogenic. Hum. Vaccin. Immunother..

[21] Wen X., Cao D., Jones R.W., Li J., Szu S., Hoshino Y. (2012). Construction and Characterization of Human Rotavirus Recombinant Vp8* Subunit Parenteral Vaccine Candidates. Vaccine.

[22] PATH Path Announces Early Closure of Pivotal Phase 3 Study of an Injectable Rotavirus Vaccine Candidate. https://www.path.org/media-center/path-announces-early-closure-of-pivotal-phase-3-study-of-an-injectable-rotavirus-vaccine-candidate/.

[23] Prashant K., Atsushi H., Christopher B., Brandy D., Soraia S., Sangeeta B.J., David B.V. (2024). Evaluating the Compatibility of three Aluminum-Salt Adjuvanted Recombinant Protein Antigens (Trivalent NRRV) Combined with a Mock Trivalent Sabin-IPV: Analytical and Formulation Challenges. Vaccines.

[24] van der Ark A.A., Hozbor D.F., Boog C.J., Metz B., Dobbelsteen G.P.v.D., van Els C.A. (2012). Resurgence of Pertussis Calls for Re-Evaluation of Pertussis Animal Models. Expert Rev. Vaccines.

[25] Jerajani K., Wan Y., Kumru O.S., Pullagurla S.R., Kumar P., Sharma N., Ogun O., Mapari S., Brendle S., Christensen N.D. (2023). Multi-Dose Formulation Development for a Quadrivalent Human Papillomavirus Virus-like Particle-Based Vaccine: Part I—Screening of Preservative Combinations. J. Pharm. Sci..

[26] McAdams D., Estrada M., Holland D., Singh J., Sawant N., Hickey J.M., Kumar P., Plikaytis B., Joshi S.B., Volkin D.B. (2022). Concordance of in Vitro and in Vivo Measures of Non-Replicating Rotavirus Vaccine Potency. Vaccine.

[27] McAdams D., Lakatos K., Estrada M., Chen D., Plikaytis B., Sitrin R., White J.A. (2021). Quantification of Trivalent Non-Replicating Rotavirus Vaccine Antigens in the Presence of Aluminum Adjuvant. J. Immunol. Methods.

[28] Coombes L., Stickings P., Tierney R., Rigsby P., Sesardic D. (2009). Development and Use of a Novel in Vitro Assay for Testing of Diphtheria Toxoid in Combination Vaccines. J. Immunol. Methods.

[29] Coombes L., Tierney R., Rigsby P., Sesardic D., Stickings P. (2012). In Vitro Antigen Elisa for Quality Control of Tetanus Vaccines. Biologicals.

[30] AlphaDiagnostics Anti-Haemophilus Influenzae, Type B (Heat Killed, Whole Bacteria) Antiserum. https://www.4adi.com/4adi/anti-haemophilus-influenzae-type-b-heat-killed-whole-bacteria-antiserum-24301-p.html.

[31] BioRad Hepatitis B Surface Antigen Ad/Ay Antibody. https://www.bio-rad-antibodies.com/results/go?w=4940-1404.

[32] HogenEsch H., Dunham A., Hansen B., Anderson K., Maisonneuve J.-F., Hem S.L. (2011). Formulation of a Killed Whole Cell Pneumococcus Vaccine—Effect of Aluminum Adjuvants on the Antibody and Il-17 Response. J. Immune Based Ther. Vaccines.

[33] Lakatos K., McAdams D., White J.A., Chen D. (2020). Formulation and Preclinical Studies with a Trivalent Rotavirus P2-Vp8 Subunit Vaccine. Hum. Vaccin. Immunother..

[34] Khatuntseva E.A., Nifantiev N.E. (2021). Glycoconjugate Vaccines for Prevention of Haemophilus Influenzae Type B Diseases. Russ. J. Bioorg Chem..

[35] Sturgess A.W., Rush K., Charbonneau R.J., I Lee J., West D.J., Sitrin R.D., Hennessey J.P. (1999). Haemophilus Influenzae Type B Conjugate Vaccine Stability: Catalytic Depolymerization of Prp in the Presence of Aluminum Hydroxide. Vaccine.

[36] Agarwal S., Hickey J.M., McAdams D., White J.A., Sitrin R., Khandke L., Cryz S., Joshi S.B., Volkin D.B. (2020). Effect of Aluminum Adjuvant and Preservatives on Structural Integrity and Physicochemical Stability Profiles of Three Recombinant Subunit Rotavirus Vaccine Antigens. J. Pharm. Sci..

[37] Kaur K., Xiong J., Sawant N., Agarwal S., Hickey J.M., Holland D.A., Mukhopadhyay T.K., Brady J.R., Dalvie N.C., Tracey M.K. (2021). Mechanism of Thimerosal-Induced Structural Destabilization of a Recombinant Rotavirus P[4] Protein Antigen Formulated as a Multi-Dose Vaccine. J. Pharm. Sci..

[38] Maman K., Zöllner Y., Greco D., Duru G., Sendyona S., Remy V. (2015). The Value of Childhood Combination Vaccines: From Beliefs to Evidence. Hum. Vaccin. Immunother..

[39] Agnolon V., Bruno C., Galletti B., Mori E., Ugozzoli M., Pergola C., O’hagan D.T., Baudner B.C. (2016). Multiplex Immunoassay for in Vitro Characterization of Acellular Pertussis Antigens in Combination Vaccines. Vaccine.

[40] Kraan H., Have R.T., van der Maas L., Kersten G., Amorij J.-P. (2016). Incompatibility of Lyophilized Inactivated Polio Vaccine with Liquid Pentavalent Whole-Cell-Pertussis-Containing Vaccine. Vaccine.

[41] WHO Thimerosal. https://www.who.int/teams/health-product-policy-and-standards/standards-and-specifications/vaccines-quality/thiomersal#:~:text=In%20some%20production%20processes%20thiomersal,of%20whole%20cell%20pertussis%20vaccine.

[42] Sawant N., Joshi S.B., Weis D.D., Volkin D.B. (2022). Interaction of Aluminum-Adjuvanted Recombinant P[4] Protein Antigen with Preservatives: Storage Stability and Backbone Flexibility Studies. J. Pharm. Sci..

[43] Dounighi N.M., Razzaghi-Abyane M., Nofeli M., Zolfagharian H., Shahcheraghi F. (2016). Study on Toxicity Reduction and Potency Induction in Whole-Cell Pertussis Vaccine by Developing a New Optimal Inactivation Condition Processed on Bordetella Pertussis. Jundishapur J. Microbiol..

[44] Biotech Panacea Easysix Package Insert. https://media.panaceabiotec.com/documents/2019/7/24/Easysix-PMPIS05903.pdf.

[45] Hexasiil Serum Institute of India. https://www.seruminstitute.com/product_hexasiil.php.

[46] Shan6 Package Insert Sanofi Healthcare. https://cdsco.gov.in/opencms/resources/UploadCDSCOWeb/2018/UploadSmPC/SmPC%20of%20%20Shan6%20Multi-dose%20of%20%20by%20Ms%20Sanofi%20Healthcare%20India%20Pvt%20Ltd%20%20%20.pdf.

[47] Sharma H., Lalwani S., Parekh S., Pujari P., Shewale S., Palkar S., Hanumante N., Gokhale S., Ks J., Kumar R. (2022). A Phase I, Open Label, Clinical Study to Assess the Safety and Immunogenicity of Indigenously Developed Liquid (Dtwp-Hepb-Ipv-Hib) Hexavalent Combination Vaccine in Healthy Toddlers Aged 16–24 Months. Hum. Vaccines Immunother..

[48] Rakesh K., Sharma I.J., Shitole A.V., Doddapaneni M., Sharma H.J. (2019). An Immunogenic Composition Having Improved Stability, Enhanced Immunogenicity and Reduced Reactogenicity and Process for Preparation Thereof.

[49] Young C.S., Ae N.Y., Ji K.E. (2017). Combination Vaccine Composition for Multiple-Dosage.

[50] ClinicalTrials.gov Study of Dtwp-Hepb-Hib-Ipv (Shan6™) Vaccine Administered Concomitantly with Routine Pediatric Vaccines to Healthy Infants and Toddlers in Thailand. https://classic.clinicaltrials.gov/ct2/show/study/NCT04429295.

[51] Hausdorff W.P., Price J., Debellut F., Mooney J., Torkelson A.A., Giorgadze K., Pecenka C. (2022). Does Anybody Want an Injectable Rotavirus Vaccine, and Why? Understanding the Public Health Value Proposition of Next-Generation Rotavirus Vaccines. Vaccines.

[52] Orsi A., Azzari C., Bozzola E., Chiamenti G., Chirico G., Esposito S., Francia F., Lopalco P., Prato R., Russo R. (2018). Hexavalent Vaccines: Characteristics of Available Products and Practical Considerations from a Panel of Italian Experts. J. Prev. Med. Hyg..

[53] WHO Requirements for Diphtheria, Tetanus, Pertussis and Combined Vaccines. https://cdn.who.int/media/docs/default-source/biologicals/vaccine-quality/requirements-for-diphteria-tetanus-pertussis-and-combined-vaccinesd5900f3c-b63e-4f7e-b7df-fcef6a149b39.pdf?sfvrsn=6da899bc_1&download=true.

[54] Xing D., Markey K., Das R.G., Feavers I. (2014). Whole-Cell Pertussis Vaccine Potency Assays: The Kendrick Test and Alternative Assays. Expert. Rev. Vaccines.

[55] Suresh K., Mahendra P.S., Vijay K.B., Ramendra P.P. (2018). Quality Control of Vaccines-a Journey from Classical Approach to 3rs. Microbiol. Curr. Res..

[56] Iwaki M., Kenri T., Senoh M. (2023). An Elisa System for Tetanus Toxoid Potency Tests: An Alternative to Lethal Challenge. Biologicals.

[57] Murakami K., Fujii Y., Someya Y. (2020). Effects of the Thermal Denaturation of Sabin-Derived Inactivated Polio Vaccines on the D-Antigenicity and the Immunogenicity in Rats. Vaccine.

[58] Agarwal S., Hickey J.M., Sahni N., Toth R.T., Robertson G.A., Sitrin R., Cryz S., Joshi S.B., Volkin D.B. (2020). Recombinant Subunit Rotavirus Trivalent Vaccine Candidate: Physicochemical Comparisons and Stability Evaluations of Three Protein Antigens. J. Pharm. Sci..

[59] Duprez J., Kalbfleisch K., Deshmukh S., Payne J., Haer M., Williams W., Durowoju I., Kirkitadze M. (2021). Structure and Compositional Analysis of Aluminum Oxyhydroxide Adsorbed Pertussis Vaccine. Comput. Struct. Biotechnol. J..

[60] Kalbfleisch K., Deshmukh S., Mei C., Ore M., Williams W., Durowoju I., Duprez J., Morin S., Carpick B., Kirkitadze M. (2019). Identity, Structure and Compositional Analysis of Aluminum Phosphate Adsorbed Pediatric Quadrivalent and Pentavalent Vaccines. Comput. Struct. Biotechnol. J..

[61] Crisel R.M., Baker R.S., Dorman D.E. (1975). Capsular Polymer of Haemophilus Influenzae, Type B. J. Biol. Chem..

[62] HogenEsch H., O’Hagan D.T., Fox C.B. (2018). Optimizing the Utilization of Aluminum Adjuvants in Vaccines: You Might Just Get What You Want. NPJ Vaccines.

[63] US-FDA Pentacel Packahe Insert. https://www.fda.gov/media/74385/download.

[64] WHO Eupenta Package Insert. https://extranet.who.int/pqweb/content/eupenta.

[65] Chem L.G. A Phase Iv Study to Assess the Safety of Eupentatm Inj. https://clinicaltrials.gov/study/NCT04056728.

[66] Meyer B.K., Ni A., Hu B., Shi L. (2007). Antimicrobial Preservative Use in Parenteral Products: Past and Present. J. Pharm. Sci..

