# Supplementary material for: Evaluating the Compatibility of New Recombinant Protein Antigens (Trivalent NRRV) with a Mock Pentavalent Combination Vaccine Containing Whole-Cell Pertussis: Analytical and Formulation Challenges
Source: Vaccines (Basel). 2024 Jun 3;12(6):609. doi: 10.3390/vaccines12060609 (PMC11209613; doi:10.3390/vaccines12060609)
Supplement: Supplementary file 1 [file vaccines-12-00609-s001.zip › vaccines-2979808-supplementary.pdf]

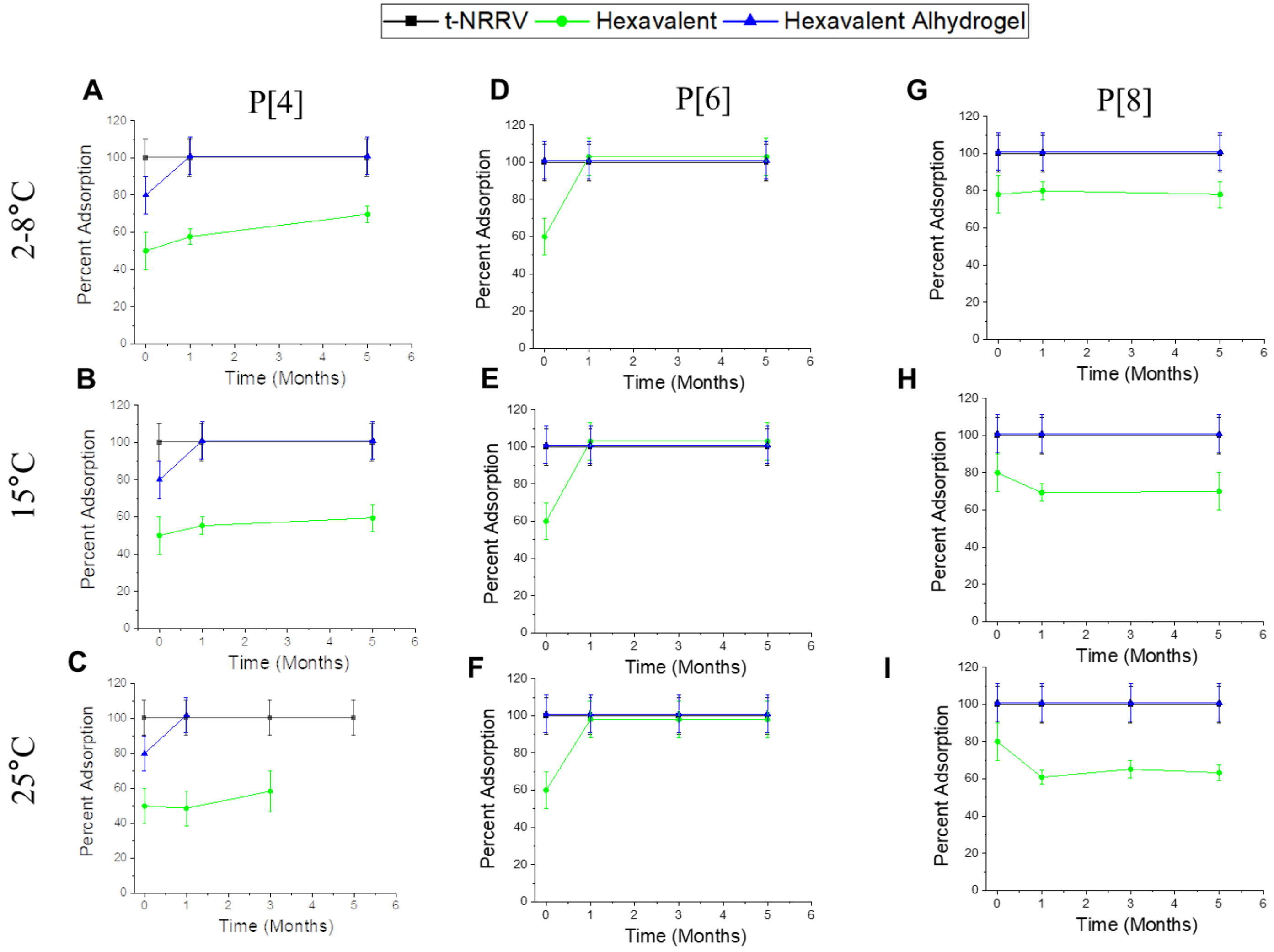

Supplemental Figure S1: Percent adsorption binding results for each of the recombinant protein NRRV P[4], P[6] and P[8] antigens formulated as a t-NRRV control (AH adjuvant only), mock hexavalent formulation (AP + AH adjuvants with t-NRRV, D, T, wP, Hib, Hep B antigens) and mock hexavalent AH formulation (AH adjuvant only with t-NRRV, D,T,wP, Hib, Hep B antigens) during storage at 2-8°C, 15°C and 25°C. Percent adsorption for P[4] antigen at 2-8°C (A), 15°C (B), and 25°C (C) in the three formulations. Percent adsorption for P[6] antigen at 2-8°C (D), 15°C (E), and 25°C (F) in the three formulations. Percent adsorption for P[8] antigen at 2-8°C (G), 15°C (H), and 25°C (I) in the three formulations. Data are presented as the mean ± range (n= 2).

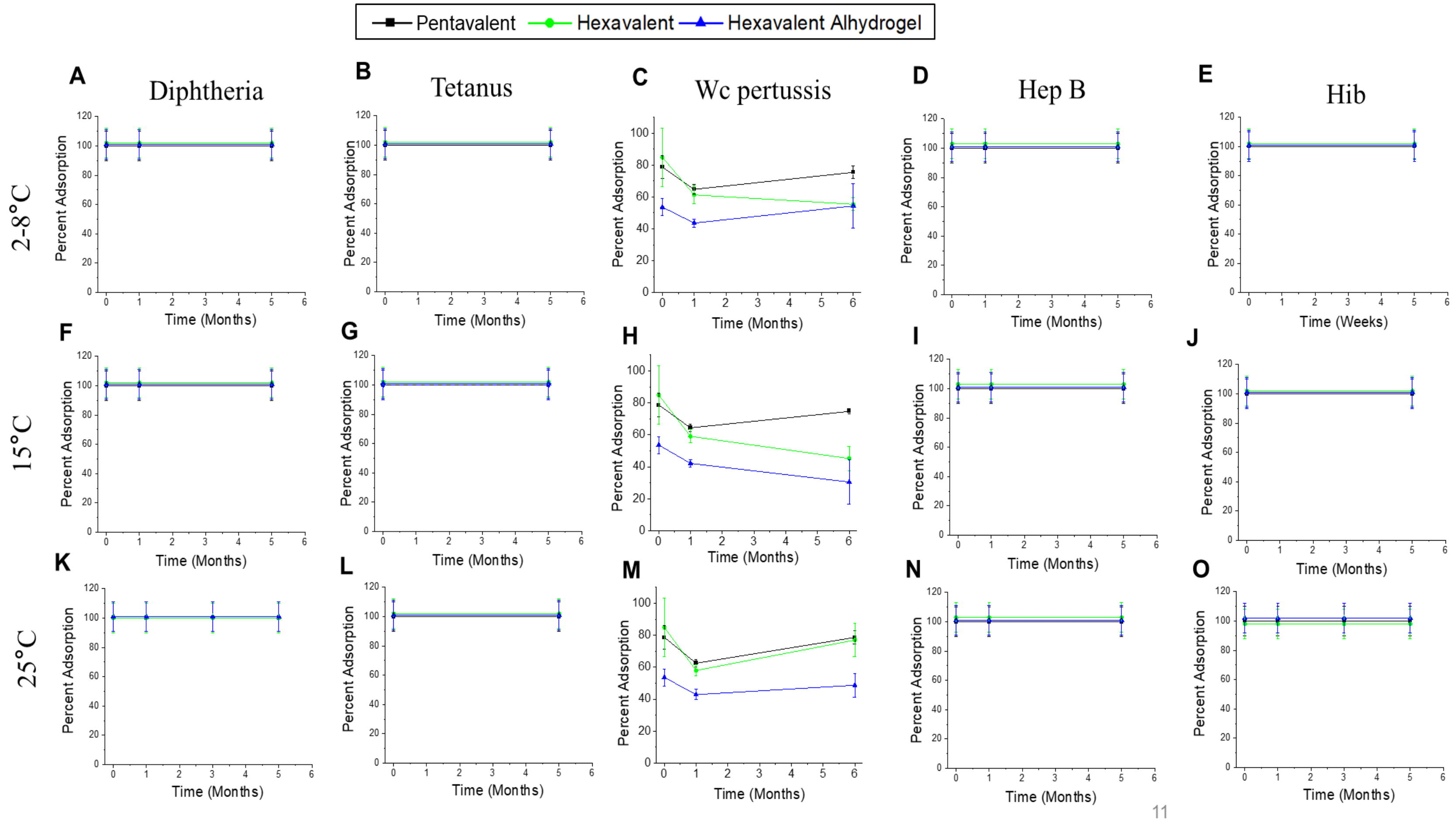

Supplemental Figure S2: Percent adsorption binding results for each of the five pentavalent antigens (D, T, wP, Hep B, Hib) formulated as a mock pentavalent control (AP + AH adjuvants with D, T, wP, Hib, Hep B antigens), mock hexavalent formulation (AP + AH adjuvants with t-NRRV, D, T, wP, Hib, Hep B antigens) and mock hexavalent AH formulation (AH adjuvant only with t-NRRV, D, T, wP, Hib, Hep B antigens) during storage at 2-8°C, 15°C and 25°C for 5 months. Percent adsorption at 2-8°C for diphtheria (A), tetanus (B), wP (C) Hep B (D) and Hib (E). Percent adsorption at 15°C for diphtheria (F), tetanus (G), wP (H) Hep B (I) and Hib (J). Percent adsorption at 25°C for diphtheria (K), tetanus (L), wP (M) Hep B (N) and Hib (O). Data are presented as the mean ± range (n= 2).
